# Sympathetic nerve–fibroblast crosstalk drives nerve injury, fibroblast activation, and matrix remodeling in pancreatic cancer

**DOI:** 10.1172/jci.insight.192814

**Published:** 2026-02-19

**Authors:** Ariana L. Sattler, Parham Diba, Kevin Hawthorne, Carl Pelz, Joe Grieco, Tetiana Korzun, Bryan Chong, M.J. Kuykendall, Rosalie C. Sears, Daniel L. Marks, Mara H. Sherman, Teresa A. Zimmers, S. Ece Eksi

**Affiliations:** 1 Cancer Early Detection Advanced Research Center (CEDAR), and; 2 Department of Cell, Developmental, and Cancer Biology, Knight Cancer Institute, Oregon Health & Science University, Portland, Oregon, USA.; 3 Department of Biomedical Engineering,; 4 Department of Molecular and Medical Genetics, and; 5 Brenden-Colson Center for Pancreatic Care, Oregon Health & Science University, Portland, Oregon, USA.; 6 Department of Pharmaceutical Sciences, College of Pharmacy, Oregon State University, Portland, Oregon, USA.; 7 Endevica Bio, Northbrook, Illinois, USA.; 8 Cancer Biology & Genetics Program, Memorial Sloan Kettering Cancer Center, New York, New York, USA.; 9 Division of Oncological Sciences, Knight Cancer Institute, Oregon Health & Science University, Portland, Oregon, USA.

**Keywords:** Cell biology, Neuroscience, Oncology, Cancer, Mouse models, Transcriptomics

## Abstract

Pancreatic cancer is a highly innervated gastrointestinal disease in which sympathetic nerves play a critical role in modulating tumor growth and the tumor microenvironment (TME). While recent studies suggest that sympathetic nerves influence various TME components, including lymphoid and myeloid immune cells, their interactions with cancer-associated fibroblasts (CAFs) remain poorly understood. CAFs are a hallmark of pancreatic tumors and are known to upregulate axon guidance and neuroactive cues, suggesting a potential feedback loop with tumor-innervating nerves. Here, we investigated the bidirectional crosstalk between sympathetic nerves and CAFs in human and mouse pancreatic tumors. Using a chemo-genetic ablation model, we selectively eliminated pancreatic sympathetic nerves and found that denervation significantly reduced tumor size in female mice. To further dissect this interaction, we established coculture systems with immortalized pancreatic fibroblasts and primary sympathetic neuron explants, identifying key transcriptional changes driven by CAF–sympathetic nerve signaling. Our findings demonstrated that sympathetic signaling enhanced CAF activation and extracellular matrix remodeling, while activated CAFs, in turn, induced transcriptional programs in sympathetic neurons associated with nerve injury response. These results establish CAFs as central mediators of the tumor-supportive role of sympathetic nerves, offering further insights into the neural regulation of pancreatic cancer progression.

## Abstract 2

We demonstrate that reciprocal signaling between sympathetic nerves and cancer-associated fibroblasts promotes nerve injury programs, fibroblast activation, and matrix remodeling in pancreatic cancer.

## Introduction

Pancreatic ductal adenocarcinoma (PDAC) is an aggressive malignancy with a dismal prognosis, sustained by a dynamic and heterogeneous tumor microenvironment (TME) that promotes tumor progression (1). PDAC is a neurotrophic cancer characterized by extensive tumor innervation (2), including sensory (3), parasympathetic (4), and sympathetic (5) nerves, which collectively contribute to disease initiation, progression, and metastasis. Growing evidence links tumor-innervating nerves to adverse clinical outcomes in PDAC (6–8). Sympathetic nerves, which regulate the “fight or flight” response, can react rapidly to acute physiological stressors and adapt to chronic conditions such as cancer (9). Current evidence suggests that sympathetic signaling and associated adrenergic receptors contribute to PDAC initiation and progression (5, 10). Sympathetic nerves begin accumulating in the early stages of pancreatic cancer, known as pancreatic intraepithelial neoplasia, and increase in density in higher-grade tumors (5). Norepinephrine, the predominant sympathetic neurotransmitter, along with other sympathetic-associated signaling, can directly induce several tumor-promoting phenotypes, including cellular metabolic reprogramming (11), proliferation (12), and invasion (7, 12). Furthermore, adrenergic signaling from sympathetic nerves directly promotes CD8^+^ T cell exhaustion and immune suppression in PDAC (13). However, the specific influences of sympathetic nerves on other components of the TME, including the extracellular matrix (ECM), endothelial cells, immune cells, and cancer-associated fibroblasts (CAFs), require further investigation. Importantly, a recent study implicates CAFs as a significant cellular partner in crosstalk with PDAC-innervating nerves (14), but the specific nature and consequences of these interactions remain unclear.

Originating from tissue-resident fibroblasts, tumor-infiltrating mesenchymal stem cells, and pancreatic stellate cells (PSCs) (15), CAFs become activated in the context of cancer and compose up to 80% of a PDAC tumor (16). Their diverse origins, as well as intratumor signaling gradients, contribute to their heterogeneity (17) and the acquisition of CAF phenotypes, including inflammatory (iCAF), myofibroblast (myCAF) (18), and antigen-presenting (apCAF) (19). Although their functions may vary with cancer stage and phenotype, CAFs directly support tumor cell growth and metabolism (20, 21), epithelial-mesenchymal transition (EMT) (22), and treatment responses by secreting tumor growth factors, chemokines, and cytokines (17). Furthermore, CAFs promote angiogenesis (23) and ECM deposition and remodeling (24), thereby contributing to PDAC progression. CAFs play a critical role in managing multiple aspects of the TME, and their role in facilitating sympathetic innervation remains under investigation. Recent work in colorectal cancer supports the significance of crosstalk between sympathetic nerves and CAFs in promoting tumor progression (25), motivating us to investigate these interactions in the CAF-rich context of PDAC.

The roles of fibroblasts and sympathetic nerves in wound healing and injury responses are well established (26, 27). However, the bidirectional interactions of fibroblasts with intratumor sympathetic nerves during PDAC progression require further investigation. Fibroblast-derived molecules, such as fibroblast growth factors (FGFs) (28) and fibroblast activation protein (FAP) (29), are typically expressed during nervous system development and can serve as axon guidance cues within developing tumors. Furthermore, a defining characteristic of PSCs, a cell of origin for PDAC CAFs, is storage of esterified retinoids (30). Retinoid signaling plays a critical role in nervous system development and maintenance (31). Similarly, other CAF-derived molecules, such as leukemia inhibitory factor (LIF) (32) and extracellular vesicles containing perineural invasion–associated transcripts (PIATs) (33), can directly contribute to neuronal remodeling in PDAC. CAFs also express a variety of axon guidance cues (34), which could contribute to intratumor nerve remodeling and exacerbate tumor-supportive phenotypes in cancer cells. For example, the upregulation of the CAF-derived axon guidance molecule SLIT2 has been linked to neuronal remodeling in PDAC (35). CAF-derived SLIT2 also binds to the ROBO1 receptor on pancreatic cancer cells, activating cortactin (CTTN) and promoting migration (36). A recent study revealed that PDAC-associated CAFs are enriched for neurotropic programs, which may support neurogenesis, neuronal differentiation, and growth (37). Interestingly, these neurotropic programs uniquely overlap with iCAF signatures, but not myCAF or apCAF.

In this study, we combined analysis of human PDAC samples, a pancreas-localized sympathectomy mouse model, and nerve-CAF coculture systems to investigate the bidirectional crosstalk between sympathetic nerves and CAFs. Comparative analysis of autonomic innervation in human and murine PDAC revealed a close spatial association between sympathetic nerves and CAFs. Using a nontoxic, pancreas-specific sympathectomy model driven by the dopamine β-hydroxylase (*Dbh*) promoter, we observed significantly reduced tumor growth in female mice. To define the transcriptional impact of sympathetic signaling on CAFs, we performed bulk RNA-seq using a coculture system, leveraging primary neonatal superior cervical ganglia (SCGs) (38). Sympathetic nerve signaling enhanced CAF activation and ECM remodeling programs, while CAFs reciprocally induced gene expression signatures associated with nerve injury responses in SCGs. Altogether, these findings reveal dynamic, bidirectional interactions between CAFs and sympathetic nerves that contribute to PDAC progression and nominate nerve-stromal crosstalk as a potential therapeutic vulnerability.

## Results

### Sympathetic innervation is abundant and is tied to the survival of PDAC patients.

**Figure 1:**
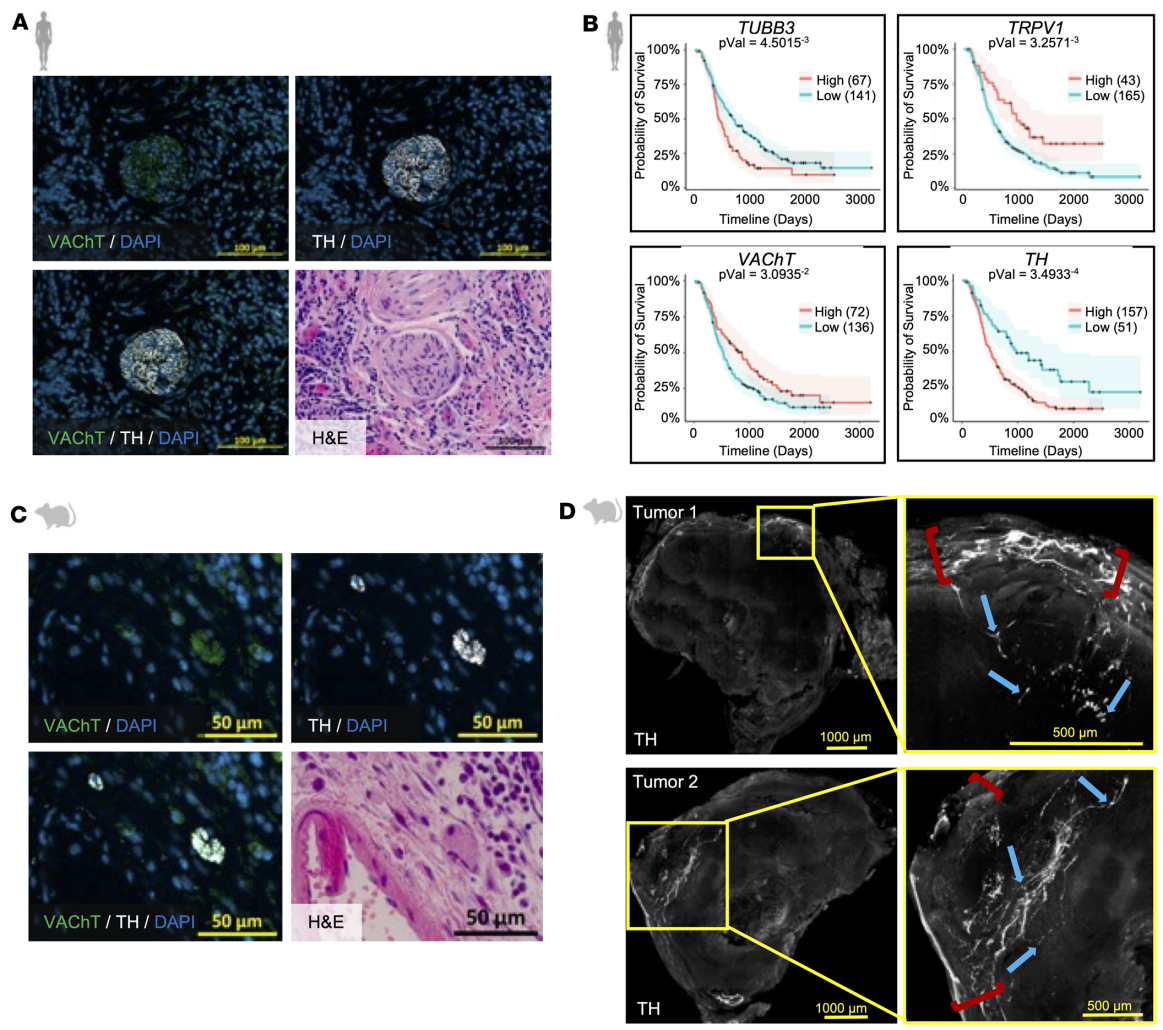
Human and murine PDAC tumors are innervated by autonomic nerves. (**A**) Co-immunofluorescent image of an intratumoral nerve bundle in human PDAC stained with VAChT (green), TH (white), and DAPI (cyan), alongside the matched H&E image. Scale bars: 100 μm. (**B**) Kaplan-Meier estimates of overall survival in patients with PDAC, stratified by primary tumor high or low gene expression of *TUBB3*, *TRPV1*, *VAChT*, and *TH* (*n* = 208). (**C**) Co-immunofluorescent image of an intratumoral nerve bundle in a murine KPC tumor stained with VAChT (green), TH (white), and DAPI (cyan), alongside the matched H&E image. Scale bars: 50 μm. (**D**) Representative cleared KPC tumors stained with TH (white). Red brackets mark tumor-encasing nerves, and blue arrows mark tumor-invading nerves. Scale bars: 500 μm and 1,000 μm.

PDAC is known to be influenced by both sympathetic-associated adrenergic (12) and parasympathetic-associated cholinergic (4) signaling. To delineate the autonomic nerve types that physically innervate PDAC, we performed immunofluorescent staining on both human and murine PDAC samples. We observed both autonomic nerve types, parasympathetic, marked by vesicular acetylcholine transporter (VAChT), and sympathetic, marked by tyrosine hydroxylase (TH), in human PDAC sections (Figure 1A and Supplemental Figure 1, A–C; supplemental material available online with this article; https://doi.org/10.1172/jci.insight.192814DS1). The spatial distribution of parasympathetic and sympathetic nerve bundles showed significant heterogeneity across distinct regions, some demonstrating a prominent VAChT^+^ parasympathetic innervation pattern and others primarily representing TH^+^ sympathetic innervation (Supplemental Figure 1A, ROI 1 and ROI 2). Intratumor nerve bundle size and localization also varied significantly (Supplemental Figure 1B, ROI 1 and ROI 2). In human tumor-adjacent pancreatic tissue, we observed a few, yet consistent nerve filaments that innervated the exocrine and endocrine areas of the pancreas (Supplemental Figure 1C, ROI 1 and ROI 2), similar to what was seen in murine tumor-adjacent tissues (Supplemental Figure 1D, ROI 1 and ROI 2).

To further evaluate the influence of intratumoral nerve-associated gene expression in PDAC, we examined the association between neuronal marker gene expression and overall survival in 208 primary PDAC tumors. These patient data originated from the recently published Oregon Pancreas Tissue Registry (OPTR) cohort (39). High expression of the pan-neuronal gene β-tubulin III (*TUBB3*) was associated with poor prognosis (Figure 1B). Elevated intratumor expression of the sensory-associated gene transient receptor potential vanilloid 1 (*TRPV1*) and the parasympathetic marker *VAChT* (human gene name: *SLC18A3*) showed positive associations with survival. In contrast, elevated *TH* expression correlated with poor prognosis (*P* < 0.05), suggesting differential effects of sensory and parasympathetic versus sympathetic innervation on PDAC survival. We further delineated these neuro-associated gene survival curves by sex and observed similar survival trends in both male and female PDAC patients (Supplemental Figure 2, A–D). We also showed that neuro-associated gene expression per PDAC stages remained relatively consistent (Supplemental Figure 2E). We further evaluated survival associated with each neuronal marker across patient stage (Supplemental Figure 2, F–J). In general, survival trends remained consistent across markers; however, the most pronounced trends were observed in patients with stage 2 disease. We also note that survival associated with high *TH* expression declined rapidly with advancing stages (Supplemental Figure 2I). These findings collectively support the hypothesis that sympathetic nerves play an instrumental role in PDAC survival.

### Murine orthotopic KPC models are innervated by sympathetic nerves.

Sympathetic nerves have been previously observed in KIC [*LSL-Kras^G12D/+^ Cdkn2a (Ink4a/Arf)^lox/lox^ Pdx1-Cre*] (5) and KPC (*Kras^LSL-G12D/+^ Trp53^LSL-R172H/+^ Pdx1-Cre*) (40) genetic mouse models of autochthonous pancreatic cancer. The KPC model, which harbors several of the most common human PDAC mutations, progresses through the established developmental stages of PDAC (41) and accurately represents the desmoplastic stroma (42). Here, we evaluated the feasibility of studying sympathetic innervation in the faster-growing orthotopic KPC model. Mouse pancreata were intentionally seeded with a low count of KPC (6419c5) (43) cells to allow sufficient time for the nerve and TME landscape to develop. Like in human PDAC, these murine orthotopic KPC tumors were innervated by VAChT^+^ parasympathetic and TH^+^ sympathetic nerves (Figure 1C). Compared with human PDAC innervation, intratumor nerve bundles in mice were typically smaller and often contained multiple separate nerve bundles near one another, suggesting decreased spatial heterogeneity in innervation. Sympathetic nerves were predominantly localized along the periphery of murine KPC tumors, with neurites often invading the tumor parenchyma (Figure 1D). We also showed similar tumor-adjacent sympathetic innervation of autocrine and paracrine areas in the KPC tumor-adjacent pancreas (Supplemental Figure 1D). Overall, these findings demonstrate that slow-growing orthotopic KPC tumors can recapitulate key features of human PDAC sympathetic innervation (5).

### CAFs surround sympathetic nerve bundles in both human and murine PDAC.

**Figure 2:**
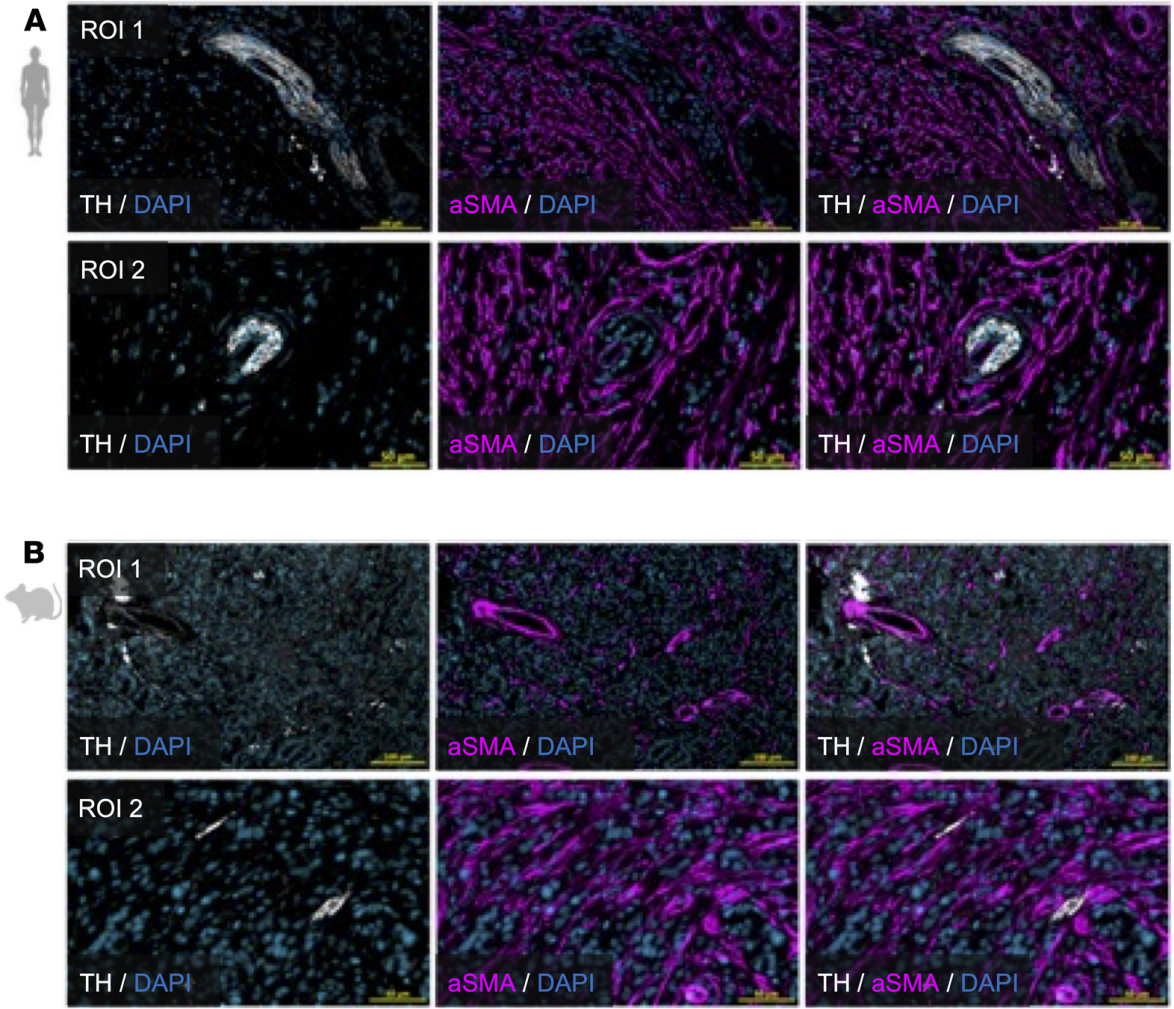
Intratumor sympathetic nerves are closely associated with CAFs. Representative co-immunofluorescent images of human (**A**) and murine (**B**) PDAC marked with TH (white), α-smooth muscle actin (aSMA) (magenta), and DAPI (cyan). Scale bars: 100 μm (top panels in **A** and **B**) and 50 μm (bottom panels in **A** and **B**).

CAFs compose a significant component of the TME of PDAC (16) and can express a variety of axon guidance cues (34–36, 44, 45). To further elucidate the spatial relationship between CAFs and sympathetic nerves within PDAC, we performed immunofluorescent staining, using TH, dopamine μ-hydroxylase (DBH), and α-smooth muscle actin (aSMA) markers on both human and murine PDAC samples. Results showed that CAFs closely surrounded sympathetic nerves, and this spatial association was conserved across human and murine PDAC tumors (Figure 2, A and B, and Supplemental Figure 1, E and F). This suggests the potential positive affinity and reciprocal signaling between tumor-innervating sympathetic nerves and CAFs.

### Generating a pancreas-localized, sympathetic-specific genetic ablation murine model.

We sought to disrupt sympathetic innervation in PDAC tumors in vivo to interrogate sympathetic-specific contributions. Current murine sympathectomy models have yielded contradictory insights, and further evidence is required to definitively determine the role of sympathetic nerves in PDAC. Two previous studies blocked sympathetic activity via systemic administration of beta blockers and reported reduced PDAC cell proliferation and improved survival in tumor-bearing mice (12, 46). Other studies have selectively ablated adrenergic and dopaminergic neurons by administering the neurotoxic chemical 6-hydroxydopamine (6-OHDA), which induces oxidative stress and, consequently, neuronal death. One study showed that orthotopically implanted pancreatic tumors were smaller after denervation (14), whereas another study demonstrated that 6-OHDA treatment led to larger pancreatic tumors and enhanced metastasis (5). To clarify these differing results, we generated a nontoxic, pancreas-specific genetic sympathectomy model that can serve as a powerful tool to delineate the specific influences of sympathetic nerves on murine PDAC tumor growth and the TME composition.

**Figure 3:**
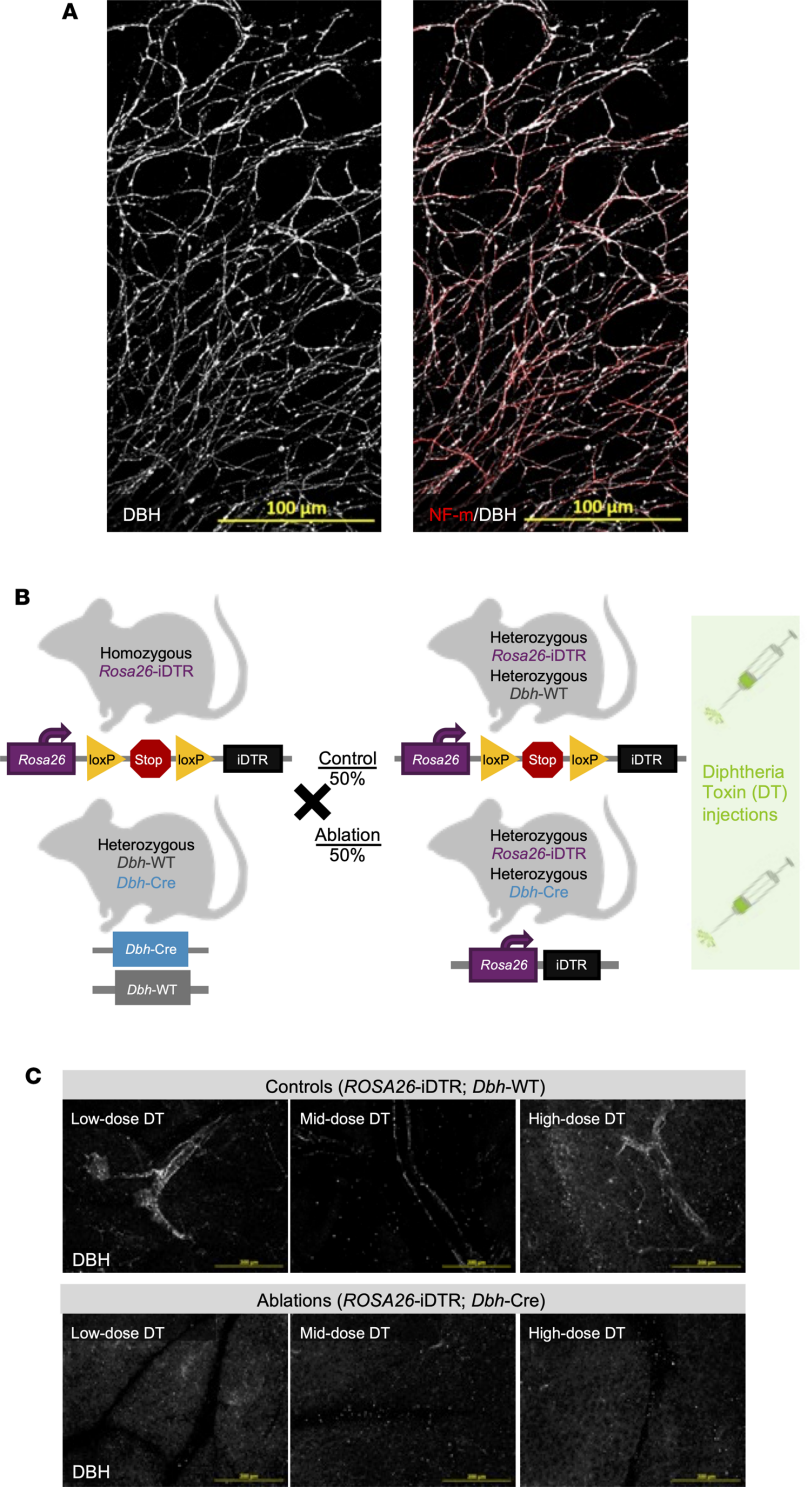
Pancreas-specific genetic ablation of sympathetic innervation. (**A**) Representative immunofluorescent image of SCG outgrowth marked with DBH (white) and neurofilament-medium (NF-m) (red). Scale bars: 100 μm. (**B**) Schematic of murine genetic sympathectomy model. Homozygous *Rosa26*-iDTR mice were crossed with heterozygous *Dbh*-Cre mice, resulting in offspring composed of 50% *Rosa26*-iDTR/*Dbh*-WT (control group) and 50% *Rosa26*-iDTR/*Dbh*-Cre (ablation group). (**C**) Representative immunofluorescent images validating healthy pancreas sympathetic ablations with low (100 ng), mid (250 ng), and high (500 ng) DT doses. Each image was acquired with the same exposure and contrast settings; maximum-intensity projections are shown. Scale bars: 200 μm.

To selectively target sympathetic innervation, we identified DBH as a sympathetic-specific marker, offering greater specificity than TH, given its downstream role in converting dopamine to norepinephrine, the primary sympathetic neurotransmitter (47). Immunofluorescent staining of ex vivo superior cervical ganglion (SCG) explant neurite outgrowth served as a robust model of sympathetic nerves. We showed consistent DBH expression throughout the extension of the axons, costained by the neurofilament-medium (NF-m) pan-neuronal marker (Figure 3A). Furthermore, we observed consistent DBH staining in both human and mouse PDAC innervation (Supplemental Figure 1, E and F). To achieve targeted sympathetic denervation, we generated an inducible chemo-genetic ablation murine model by crossing a heterozygous *Dbh*-Cre (B6.Cg-*Dbh^tm3.2(cre)Pjen^*/J) mouse with a homozygous *Rosa26*-iDTR [C57BL/6-*Gt(ROSA)26Sor^tm1(HBEGF)Awai^*/J] mouse line (Figure 3B). The resulting cross produced half of the offspring expressing the diphtheria toxin receptor (DTR), specifically in sympathetic neurons, rendering them susceptible to ablation upon exposure to diphtheria toxin (DT). The remaining half of the offspring served as controls, lacking Cre and therefore the DTR expression and thus not susceptible to DT-induced ablation. With this model, all mice received several intrapancreatic DT injections to ensure consistency across cohorts and to control for off-target effects of DT or potential injection-related inflammation.

Intrapancreatic DT treatment demonstrated a robust and consistent reduction of DBH expression in cleared and stained healthy pancreas tissues (Figure 3C). We observed a consistent reduction in DBH staining across low, mid, and high DT doses. Both male and female cohorts were analyzed, and tissues were evaluated 1 month after ablation to ensure that the ablations remained consistent for the duration of our slow-growing orthotopic tumor model.

**Figure 4:**
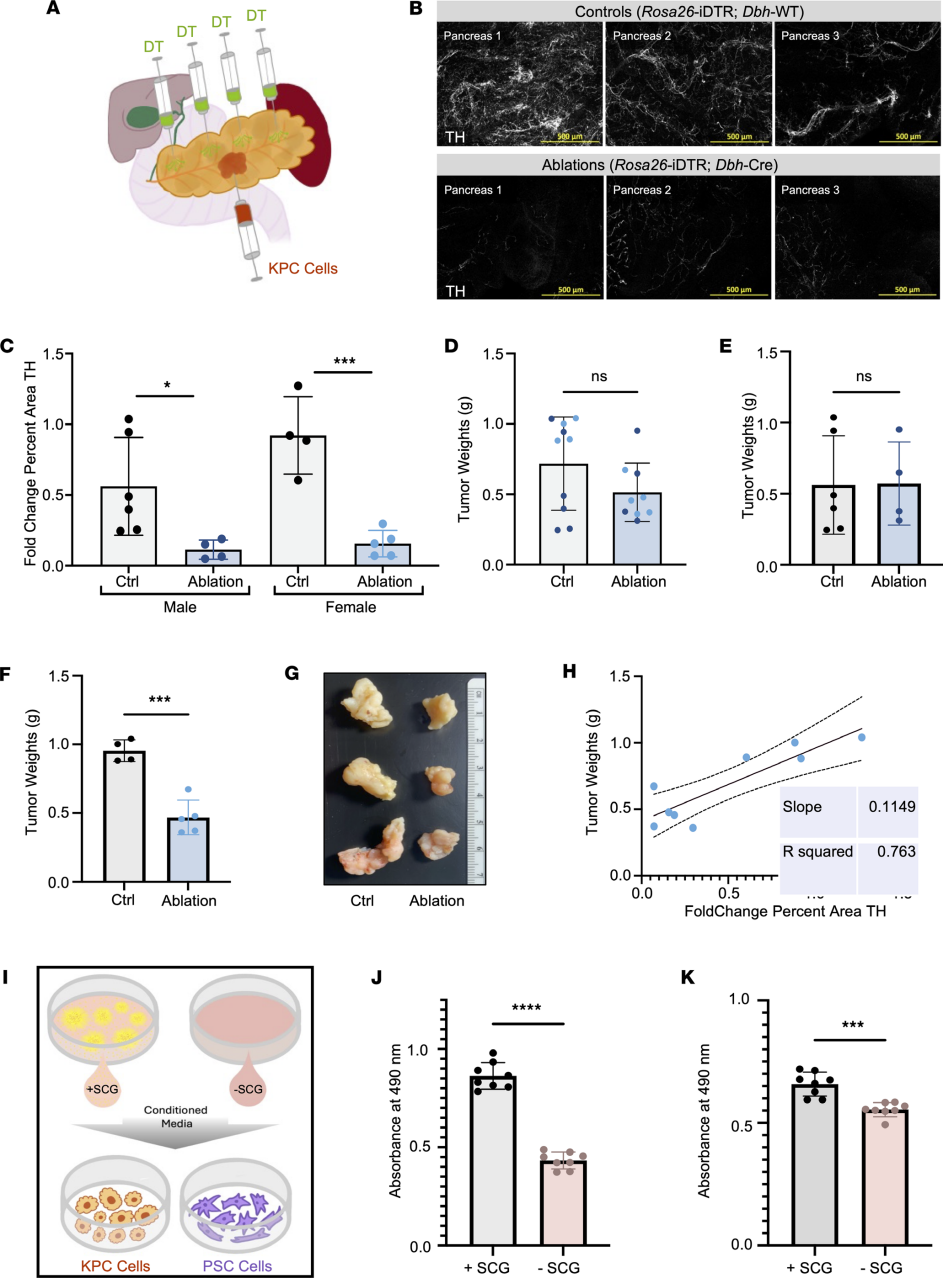
Sympathetic nerves promote female PDAC tumor growth. (**A**) Schematic of intrapancreatic DT injections combined with an orthotopic KPC (6419c5) injection. (**B**) Representative immunofluorescent staining of sympathetic nerves with TH (white) in the tumor-adjacent pancreas of control versus sympathectomized cohorts. Images were acquired with the same exposure and contrast settings; maximum-intensity projections are shown. Scale bars: 500 μm. (**C**) Quantification of normalized TH percent area (fold change) of control versus sympathectomized tumor-adjacent pancreas in male (*n* = 6 control, *n* = 4 ablation) and female (*n* = 4 control, *n* = 5 ablation) mice. Data were normalized to the mean of the control groups across staining batches. Each dot represents 1 animal, bar plot mean ± SD; *P < 0.05, ***P < 0.001; paired Wilcoxon’s signed rank test. (**D**) Combined male (dark blue) and female (light blue) tumor weights (grams) of control and sympathetic ablation groups. Each dot represents 1 animal, mean ± SD; not significant (ns) *P* > 0.05; paired Wilcoxon’s sig ned rank test. (**E** and **F**) Endpoint KPC tumor weights (grams) separated by sex: male (*n* = 6 control, *n* = 4 ablation) (**E**) and female (*n* = 4 control, *n* = 5 ablation) (**F**). Each dot represents 1 animal, bar plots mean ± SD; ***P < 0.001; unpaired, 2-tailed Student’s *t* test. (**G**) Representative endpoint tumors of female control and ablated tumors. The tape measure is marked in centimeters. (**H**) Correlation between female normalized TH percent area and KPC orthotopic tumor weights (grams). (**I**) Schematic of conditioned medium proliferation assay. (**J** and **K**) Proliferation of KPC (6419c5) (**J**) and PSC (mPSC1) (**K**) cells cultured with SCG-conditioned (+SCG) or control (–SCG) medium. *n* = 8 replicates; bar plots mean ± SD; ***P < 0.001, ****P < 0.0001; unpaired, 2-tailed Student’s *t* test.

Next, we applied our validated inducible denervation method to study the effects of sympathetic nerve ablation in our syngeneic orthotopic KPC tumor model. We administered 4 intrapancreatic injections of DT, immediately followed by a KPC (6419c5) cell injection (Figure 4A). Simultaneous administration was key to reducing additional stress and off-target effects and allowing the KPC tumors to initiate and progress in a sympathetically depleted pancreas. To confirm the efficacy of these intrapancreatic DT administrations, we performed immunofluorescent staining for TH expression in tumor-adjacent pancreata (Figure 4B), and normalized quantification revealed a significant reduction in sympathetic nerve density in both male and female ablated cohorts (Figure 4C). In addition to validating DBH depletion in the tumor-adjacent pancreas, we demonstrated that there was no change in TH expression in the colons, lungs, and hearts of mice in either cohort of our model (Supplemental Figure 3, A–C). We also showed representative images of male and female intratumor innervation in control and ablated animals (Supplemental Figure 3D). Furthermore, systemic health metrics, including body weight, inflammation-induced spleen changes, and glucose levels, were assessed to ensure that sympathetic ablation did not cause significant physiological distress (Supplemental Figure 3, E–H). Overall, we validated that this approach led to precise, inducible elimination of sympathetic nerves.

### Pancreas-specific sympathectomy reduced tumor size in female mice.

Despite effective pancreatic sympathectomy, initial analysis of tumor size across all samples did not show significant differences between control and denervated groups (Figure 4D). However, when stratified by sex, a distinct pattern emerged: male tumor weights remained unchanged (Figure 4E), whereas female tumor weights and sizes were significantly reduced by sympathectomy (Figure 4, F and G). Further analysis revealed a positive correlation between nerve density and tumor weight in females, suggesting a potential role for sympathetic signaling in female-specific tumor progression (Figure 4H). These results indicated that sympathetic denervation selectively impacted tumor growth in female but not male mice in our KPC model, suggesting a potential sex-dependent influence of adrenergic signaling on PDAC progression (Supplemental Figure 3I).

Given our observed differences in tumor size and the known role of adrenergic signaling in promoting PDAC cell proliferation (12), we evaluated the proliferative effects of sympathetic nerve stimulation. Using the primary SCG explant model, we assessed the impact of potential paracrine adrenergic signaling on murine KPC cancer cells and CAFs by measuring proliferation (Figure 4I). SCG-conditioned medium significantly enhanced the proliferation of KPC cell line 6419c5 cells, the same line used in our orthotopic model (Figure 4J). To investigate the effect of SCG paracrine signaling on CAF proliferation, we used the mPSC1 (PSC) cell line. This PSC cell line was derived from murine pancreatic tissue, becomes activated upon plastic adherence, and shows significant heterogeneity (20, 30), thus providing a biologically relevant framework for in vitro CAF studies. Our results demonstrated that SCG-conditioned medium also significantly enhanced PSC cell proliferation (Figure 4K). These findings suggest that sympathetic nerves simultaneously promote the proliferation of tumor cells and CAFs, which may explain the increased tumor growth observed in control mice compared with sympathectomized mice.

### SCG signaling activates CAF genes associated with poor patient outcomes.

**Figure 5:**
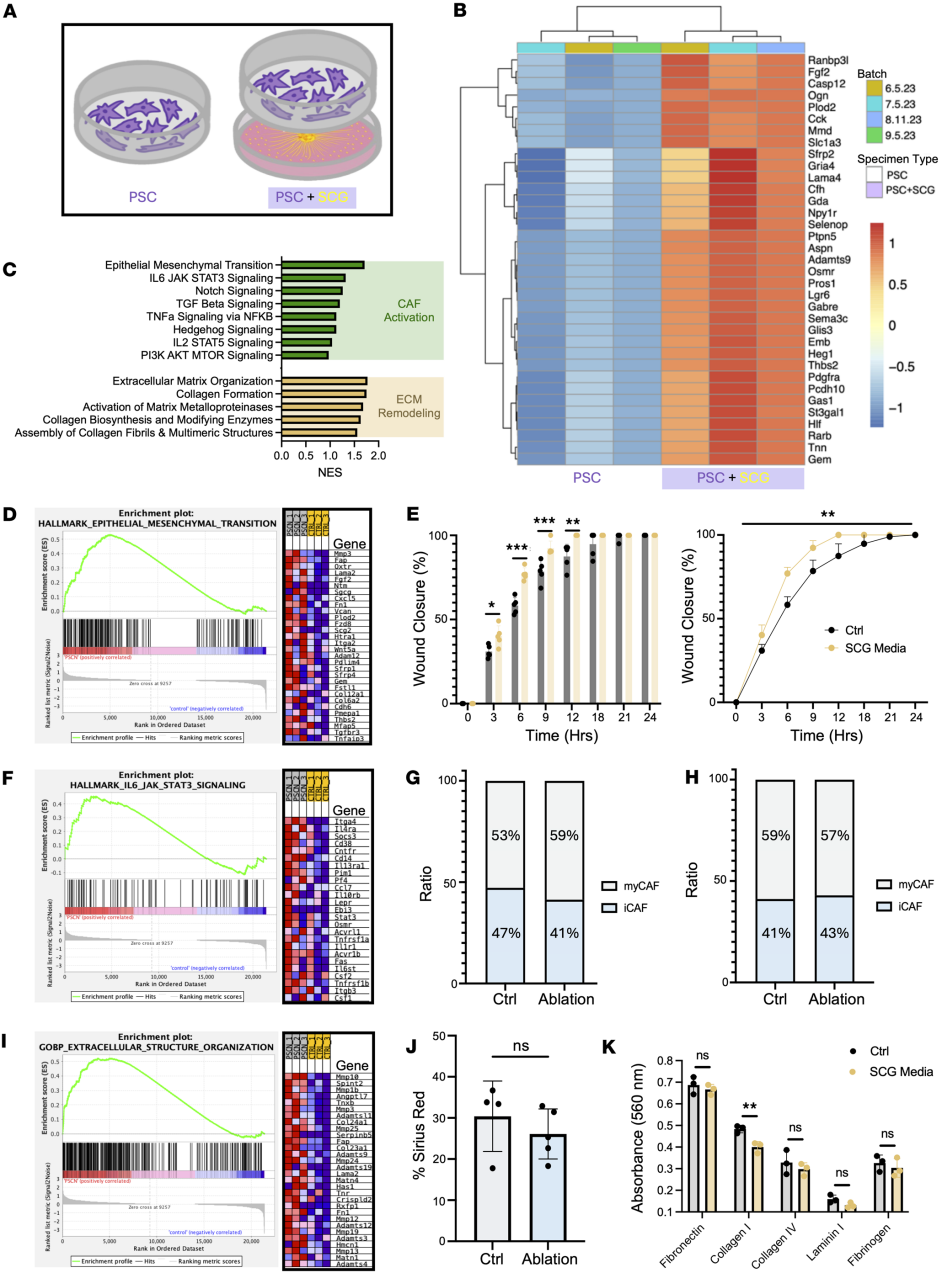
Sympathetic nerves drive CAF activation and ECM remodeling. (**A**) Schematic of indirect coculture of PSCs with SCGs. (**B**) Heatmap of all differentially expressed protein-coding genes in PSC controls compared with PSC+SCG experimental groups. (**C**) Gene set enrichment analysis (GSEA) normalized enrichment scores (NES) of signaling pathways related to CAF activation (hallmarks) and extracellular matrix remodeling (GO biological processes). (**D**) Epithelial-mesenchymal transition GSEA plot (NES = 1.71, FDR *q* value = 0.007) and top 30 significantly enriched genes. (**E**) Wound healing assay, percentage bar plot, and closure curve of PSCs treated with control (Ctrl) and SCG-conditioned medium. *n* = 5 wounds per time point, per treatment; mean ± SD; *P* > 0.05, *P < 0.05, **P < 0.01, ***P < 0.001; unpaired, 2-tailed Student’s *t* test; 2-way ANOVA, repeated measures. (**F**) IL-6/JAK/STAT3 signaling GSEA plot (NES = 1.32, FDR *q* value = 0.046) and top 25 significantly enriched genes. (**G** and **H**) Quantified ratio percentage of iCAFs to myCAFs in Ctrl and ablated tumors of female (**G**) and male (**H**) mice. (**I**) Extracellular structure reorganization GSEA plot (NES = 1.74, FDR *q* value = 0.19) and top 25 significantly enriched genes. (**J**) Quantification of ECM (percent area) in female tumors. Each dot represents 1 animal; *n* = 9; bar plot mean ± SD; ns *P* > 0.05. (**K**) Adhesion assay of PSCs treated with Ctrl medium and SCG-conditioned medium. Bar plot. *n* = 3 wells per ECM scaffolding; mean ± SD; ns *P* > 0.05, **P < 0.01; unpaired, 2-tailed Student’s *t* test.

Although CAFs regulate a variety of axon guidance cues, including Slit2 (35, 36), Sema3a (44), Sema3d (45), Netrin-1 (48), and Netrin-G1 (21), it remains unclear whether CAFs actively respond to sympathetic signaling. Using an indirect SCG explant coculture system with activated PSCs, we investigated the transcriptional effect of sympathetic signaling on activated PSCs (Figure 5). We separated SCG explants from PSCs using Transwell inserts, cocultured for 70 hours (Figure 5A), and performed bulk RNA-seq to identify differentially expressed genes in PSCs upon exposure to sympathetic-derived signaling. PSCs exposed to SCG-derived adrenergic signaling significantly upregulated 35 protein-coding genes (log_2_ fold change ≥ 0.5, adjusted *P* ≤ 0.05) (Figure 5B and Supplemental Figure 4, A–C). Upon initial evaluation, several upregulated genes, including *GAS1*, *GABRE*, *LAMA4*, *MMD*, *OSMR*, *PLOD2*, and *ST4GAL1*, were associated with poorer prognosis in the human OPTR PDAC cohort (Supplemental Figure 4D).

### SCG signaling drives CAF activation and ECM remodeling.

To further characterize PSC transcriptional shifts induced by sympathetic signaling, we performed gene set enrichment analysis (GSEA). A query of several upregulated pathways revealed a strong association with CAF activation and ECM remodeling (Figure 5C). EMT-related genes were strongly enriched, with the top 30 genes including *Fap*, *Fgf2*, *Cxcl5*, and *Wnt5a* (Figure 5D). Functionally, we showed that SCG-conditioned medium increased CAF invasion (Figure 5E). We also observed significant enrichment of IL-6/JAK/STAT3 signaling (Figure 5F), consistent with a shift toward an iCAF phenotype. Using the sympathetic ablation model, we evaluated the iCAF and myCAF states in response to sympathetic input. We showed that the tumors derived in female mice, which showed a significant reduction in tumor size in response to sympathectomy, had a 6% lower iCAF to myCAF ratio, whereas male-derived tumors had approximately the same ratios (Figure 5, G and H, and Supplemental Figure 5, A–D). Additionally, PSCs treated with SCG medium significantly upregulated iCAF-associated cytokines (Supplemental Figure 5, E and F).

We also identified strong enrichment for ECM remodeling pathways (Figure 5, C and I), suggesting that sympathetic nerves could influence CAF function and ECM deposition, which have previously been shown to support cancer progression and metastasis (49). Furthermore, genes enriched in extracellular structure organization revealed a broad set associated with matrix deposition, degradation, and reorganization. We evaluated ECM in the in vivo sympathetic ablation model and found a subtle yet non-significant decrease in ECM density in ablated tumors of female mice (Figure 5J and Supplemental Figure 5, G–I). Collagen fiber alignment, coherency, and orientation were unchanged between control and ablated tumors (Supplemental Figure 5, J and K). When PSCs were treated with SCG medium in vitro, PSCs were significantly less adherent to collagen I as compared with fibronectin, collagen IV, laminin I, and fibrinogen (Figure 5K), which supports the findings of SCG-induced ECM remodeling in PSCs and also the iCAF phenotypic shift.

### PSC-derived SEMA3C expression in response to sympathetic signaling enhances PDAC EMT.

**Figure 6:**
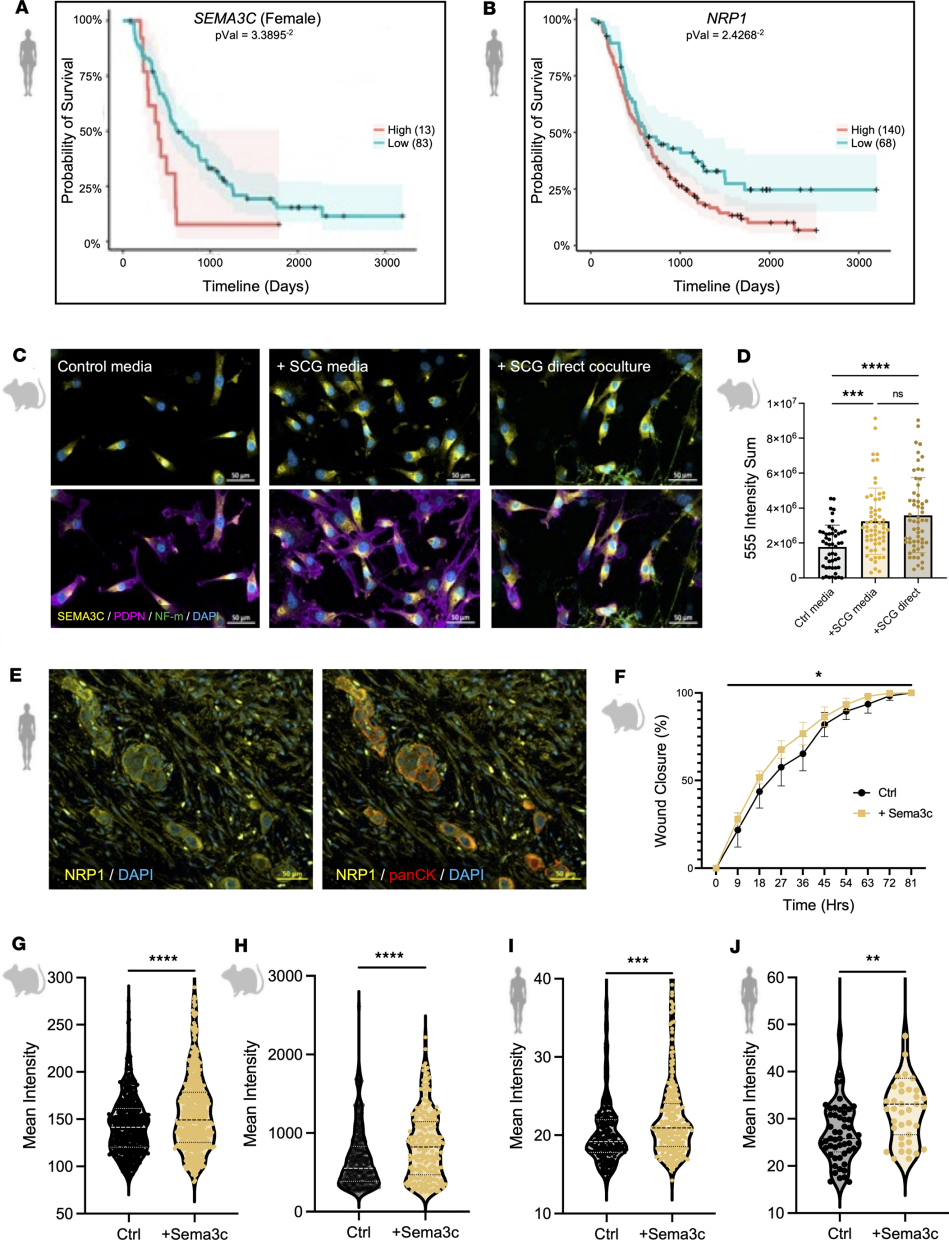
Upregulation of Sema3c enhances metastatic phenotypes. (**A** and **B**) Kaplan-Meier estimates for female PDAC patients stratified by *SEMA3C* (**A**) and *NRP1* (**B**) expression. (**C**) Co-immunofluorescent images of SEMA3C (yellow), PDPN (magenta), and NF-m (green) expressed in PSCs cocultured with SCG medium or SCGs directly. Scale bars: 50 μm. (**D**) Intensity sum of SEMA3C expression in PSCs cocultured with control medium, SCG-conditioned medium, and directly with SCGs. Each dot represents 1 cell, bar plot mean ± SD; ns *P* > 0.05, ***P < 0.001, ****P < 0.0001. (**E**) Immunofluorescent staining of NRP1 (yellow) in a human PDAC tumor section. Scale bars: 50 μm. (**F**) Wound healing assay percent closure of KPC (6419c5) cells treated with Ctrl medium and recombinant SEMA3C. *n* = 4 wounds per time point, per treatment; mean ± SD; *P < 0.05; 2-way ANOVA, repeated measures. (**G**–**J**) Mean intensity quantification of immunofluorescent staining of KPC (6419c5) cells with SNAIL/SLUG (**G**) and Vimentin (**H**) and Panc1 cells with SNAIL/SLUG (**I**) and Vimentin (**J**).

In addition to CAF-related pathways, we identified 6 genes (18% of the total significantly upregulated genes) associated with cell-cell attachment and nerve-related signaling (Supplemental Figure 6A): *Cck*, *Sema3c*, *Gria4*, *Slc1a3*, *Gas1*, and *Pcdh10*. Although CAFs have been shown to inherently express neuro-associated genes (28, 34), our results suggest that sympathetic signaling may amplify this neuro-associated gene expression in CAFs, potentially driving a feedforward loop of nerve-stroma crosstalk within the TME that influences PDAC tumors. The axon guidance cue semaphorin 3c (SEMA3C) emerged as a key candidate, as high intratumor *SEMA3C* expression was strongly associated with poor survival in female patients with PDAC (Figure 6A and Supplemental Figure 6B). We also noted that the expression of the canonical receptors for SEMA3C, NRP1, and NRP2 was associated with poor survival in PDAC patients (Figure 6B and Supplemental Figure 6C). We validated that PSC cells significantly upregulated SEMA3C in both indirect (+SCG medium) and direct (+SCG direct) cocultures with SCGs (Figure 6, C and D). We also found that NRP1 was significantly upregulated in murine KPC tumor tissue, as compared with the tumor-adjacent pancreas (Supplemental Figure 6D), and that NRP1 was expressed in both well-differentiated and poorly differentiated regions of these KPC tumors (Supplemental Figure 6E). We further observed that NRP1 was expressed by both cancer cells and fibroblasts in murine KPC tumors (Supplemental Figure 6E) and human PDAC (Figure 6E).

Previous literature has identified SEMA3C as a contributor to EMT in pancreatic (50), prostate (51), and breast cancers (52); therefore, we further evaluated ECM-associated shifts. The treatment with recombinant SEMA3C resulted in an increase in PSC proliferation (Supplemental Figure 6F) and a significant increase in KPC cell invasion (Figure 6F). We also confirmed that both SNAIL/SLUG and Vimentin expression was increased in both murine KPC (Figure 6, G and H) and human Panc1 (Figure 6, I and J) cells upon treatment with recombinant SEMA3C. We note that recombinant mouse SEMA3C also altered PSC cytokine profiles toward an iCAF-like state, recapitulating the effects of sympathetic neuron–derived SCG medium (Supplemental Figure 5, E and F).

### KPC cells and activated fibroblasts enhance axon outgrowth.

**Figure 7:**
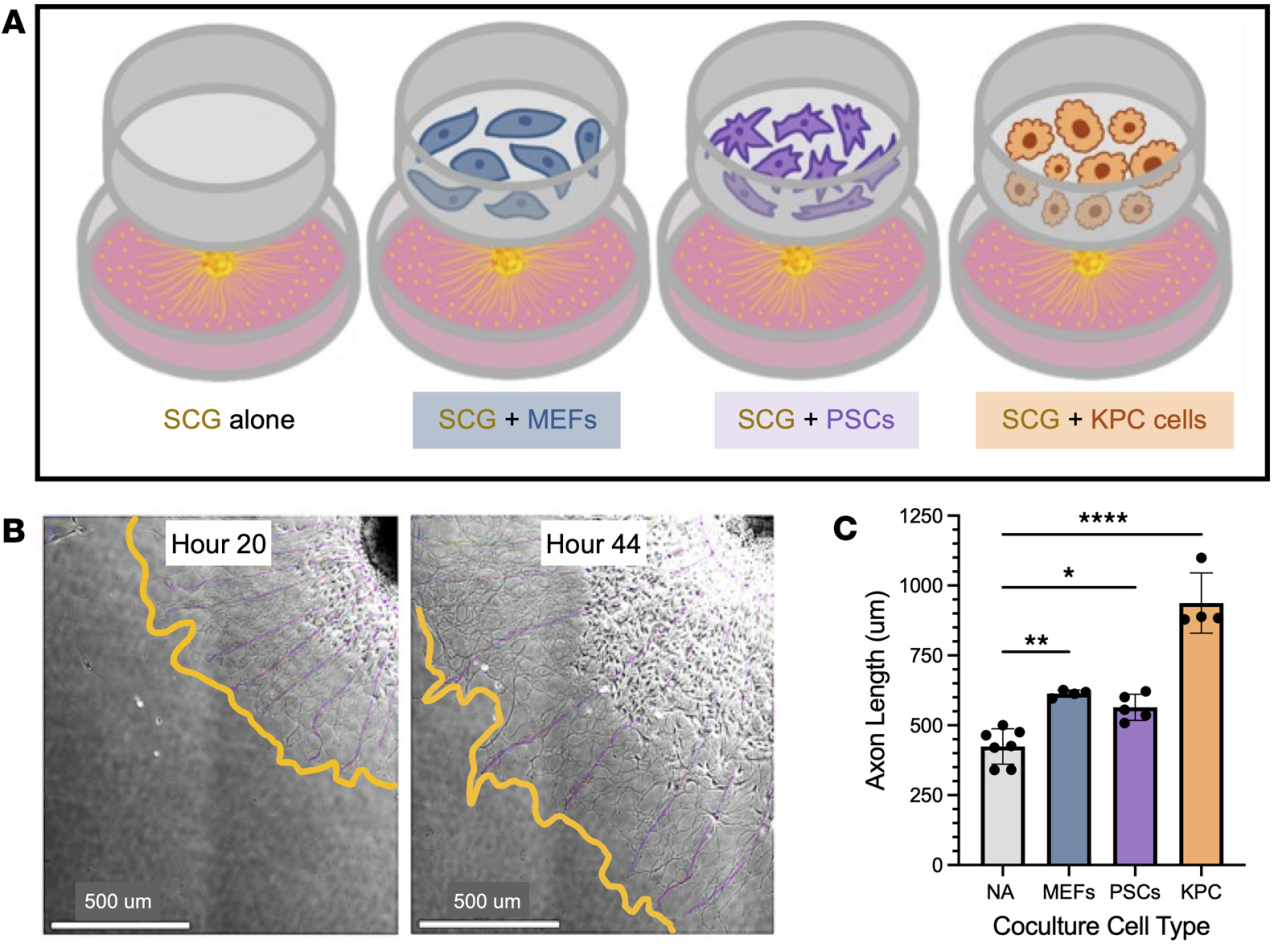
Cancer and fibroblast signaling enhances SCG axon outgrowth. (**A**) Schematic of indirect coculture systems. SCG explants (yellow) were plated on the bottom of each well (the schematic does not represent the actual number of SCG explants, typically around 8 explants per well). SCG explants were cocultured either alone (no cells) or with cells on inserts, including MEFs (control fibroblasts, blue), mPSC1 cells (pancreas-derived CAFs, violet), and 6419c5 KPC cells (orange). (**B**) Representative phase-contrast image of SCG explant radial axon outgrowth at hour 22 and hour 44 after plating. The yellow outline indicates the edge of measured averaged radial axon outgrowth, and purple represents average axon traces. Scale bars: 500 μm. (**C**) Quantification of SCG axon length of explants in coculture conditions: NA (no cells), MEFs (Ctrl fibroblasts), PSCs (mPSC1), and KPC (6419c5) cells. Each dot represents one SCG explant, mean ± SD; *P < 0.05, **P < 0.01, ****P < 0.001; 1-way ANOVA.

Neuronal cell bodies are located outside the target organ, posing challenges for capturing transcriptional changes in nerves in response to cancer growth and stromal changes. A recent study used retrograde tracing to sequence pancreatic tumor–innervating nerves, identifying CAFs as potential partners in the PDAC TME (14). To model sympathetic axon outgrowth and transcriptomic changes that are induced by CAFs, we leveraged our SCG explant Transwell coculture model with activated PSCs. Our SCG explant model uniquely preserves several components of the ganglion structure and microenvironment, including glial cells, which play an essential role in neuronal maintenance and function. SCG explants were cocultured in Transwells with control fibroblast (mouse embryonic fibroblasts [MEFs], NIH 3T3 cells), activated PSC (mPSC1), or KPC (6419c5) cells (Figure 7A). Axon extension was captured using phase-contrast microscopy over a 24-hour period to quantify neuronal outgrowth (Figure 7B). Neurite extensions emanating from each individual SCG explant were traced and averaged across coculture systems. As expected, SCG explants cocultured with our positive control, KPC cells, which secrete a variety of neural growth factors, significantly increased axon outgrowth (Figure 7C). SCG explants cocultured with activated MEFs and PSCs resulted in only a marginal increase in axon extension. We also showed increased axon outgrowth rates in SCG explants cocultured directly and indirectly with activated PSCs (Supplemental Figure 7, A and B) and preferential growth toward PSCs within a Matrigel dome (Supplemental Figure 7, C and D). These observations suggest that fibroblasts might exert more subtle phenotypic effects on sympathetic nerve outgrowth.

### Activated PSCs induce injury response in SCG explants.

**Figure 8:**
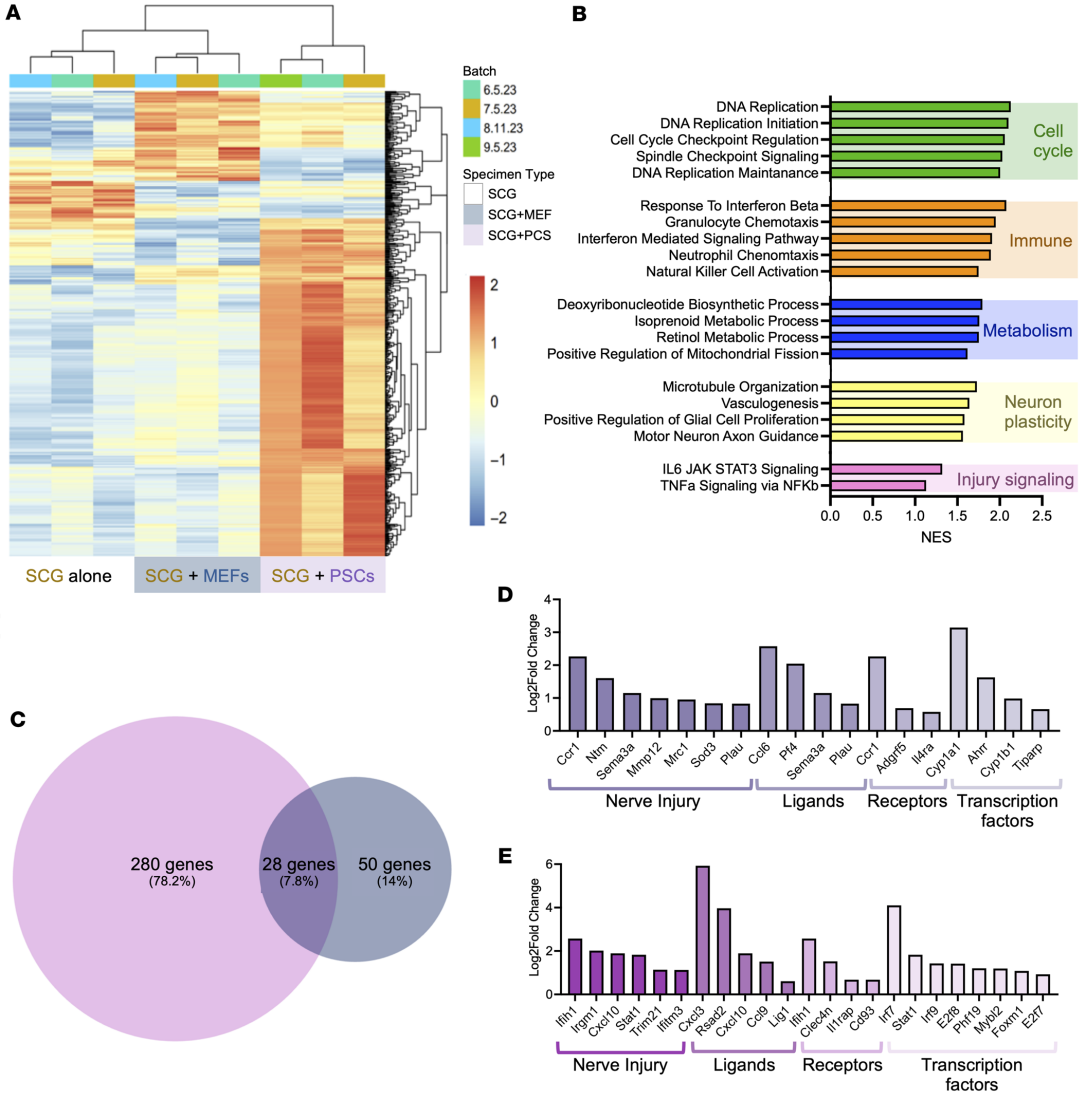
CAF-specific transcriptional effects on SCG explants. (**A**) Heatmap of differentially expressed genes comparing SCGs alone (yellow), SCGs cocultured with MEFs (blue), and PSC cells (purple). The genes shown (rows) are all significantly differentially expressed genes across all condition comparisons. (**B**) GSEA of SCG+PSC versus SCG of biological processes enriched gene sets. From top to bottom, select pathways upregulated: cell cycle (green), immune response (orange), metabolic pathways (blue), neuron plasticity (yellow), and injury signaling (pink). (**C**) Venn diagram of upregulated genes of different coculture comparisons. (**D** and **E**) Bar graphs delineating specific genes upregulated in both SCG+PSC and SCG+MEF samples (**D**) and SCG+PSC samples (**E**), categorized by nerve injury, ligands, receptors, and transcription factor (log_2_ fold change).

To investigate the impacts of MEFs versus activated PSCs on SCG transcription, we sequenced SCG explants following coculture. We identified several protein-coding genes that distinctly shifted under culturing conditions, with MEFs inducing fewer transcriptional changes than activated PSCs (Figure 8A and Supplemental Figure 7, E and F). GSEA revealed significant enrichment in several categories previously shown to be related to nerve injury responses, such as cell cycle (53), immune response (54), metabolism (55), neuron plasticity (56), and select signaling pathways associated with injury response (57) in the setting of PSC coculture (Figure 8B). We also note that MYC signaling (58), mTORC1 (59), IL-6/JAK/STAT3 signaling (60), and EMT (61) were also enriched and are associated with peripheral nerve injury response (Supplemental Figure 7G). These results suggest that exposure to PSC-derived CAFs further exacerbates the transcriptional response to sympathetic nerve injury.

### Activated PSCs reprogram sympathetic nerve transcriptional programs toward a sustained injury response.

Beyond shifts in injury-associated transcription, we observed that activated PSCs induced greater changes in gene expression than MEFs, with only 28 protein-coding genes (7.8%) shared between conditions (Figure 8C). In contrast, 280 genes (78.2%) were significantly upregulated in response to activated PSCs, indicating a more profound impact of activated PSCs on SCG transcriptional programs.

Upon examination of the overlapping genes enhanced by both fibroblast cell lines, we identified several genes for nerve injury as well as ligands, receptors, and transcription factors that may influence sympathetic neuron phenotypes. Of the 28 overlapping SCG genes enhanced by both PSCs and MEFs, *Ccr1*, *Ntm*, and *Sema3a* stood out as genes associated with nerve injury, while several ligands, such as *Ccl6*, *Pfa*, *Sema3a*, and *Mmp12*, and receptors, such as *Cyp1a1* and *Il4ra*, were upregulated (Figure 8D and Supplemental Figure 7H). Upon closer examination of the 280 genes uniquely upregulated when SCGs were cocultured with PSCs, we identified several additional genes that may contribute to a sustained injury response in sympathetic neurons. The nerve injury–associated genes included *Ifih1*, *Cxcl10*, *Stat1*, and *Trim21* (Figure 8E). Furthermore, upregulated ligands included *Cxcl3*, *Cxcl10*, and *Ccl9*, while upregulated receptors included *Irih1*, *Clec4n*, and *Il1rap*. Differentiating these unique gene changes provides insights into the general transcriptional shifts induced by activated fibroblasts and the pancreas-specific CAF-induced shifts. We also highlight that each receptor or ligand has binding potentials and that several genes encoding proteins, such as *Ccl6* (62), *Pf4* (63), *Sema3a* (44), *Cxcl3* (64), *Cxcl10* (65), and *Ccl9* (66), have been shown to have broader tumor-supportive roles in PDAC.

## Discussion

Sympathetic influences are increasingly recognized to have roles in tumor initiation, progression, and recurrence in PDAC (10, 12, 46, 48). To overcome the limitations of current mouse models of sympathetic innervation in PDAC, we developed a novel genetic sympathectomy KPC model to elucidate the direct contributions of sympathetic nerves to PDAC progression and to changes in the TME, particularly CAFs. We further identified specific transcriptional programs leveraging ganglion explant coculture systems, in which we observed that adrenergic signaling induced CAF activation and ECM remodeling, while CAF signaling exacerbated sympathetic injury response. Our results provide further understanding of the specific reciprocal interactions between sympathetic nerves and CAFs in PDAC tumors.

Previous chemical sympathectomy strategies used in PDAC have yielded seemingly contradictory results and the potential for off-target effects (5, 14, 46, 67, 68). Systemic inhibition of sympathetic signaling using beta blockers has been shown to reduce PDAC proliferation and improve survival (12, 46). In contrast, studies using chemical sympathectomy via 6-OHDA have reported divergent outcomes: one study showed decreased tumor sizes (14), whereas another observed larger tumors and increased metastasis (5). Several biological factors may account for these opposing findings. Unlike beta blockade, which functionally inhibits adrenergic signaling, 6-OHDA induces neuronal death through dopamine and norepinephrine transporter–mediated uptake and ROS-driven mitochondrial injury, introducing oxidative stress that may also alter the pancreatic microenvironment. Systemic administration or diffusion of the chemical may also affect off target organs as well as differentially influencing stromal remodeling, immune composition, and tumor-nerve dynamics, contributing to disparate outcomes across studies. In addition, the timing of denervation, whether performed during early- or late-stage tumor evolution or at differing ages and therefore interacting with developmental or hormonal differences, may further contribute to variable outcomes. These complexities motivated our development of a nontoxic, pancreas-localized chemo-genetic sympathectomy model, which enabled us to define the direct role of sympathetic nerves in PDAC independent of systemic or ROS-related effects.

Our novel chemo-genetic sympathectomy model is nontoxic, sympathetic-specific, and pancreas-localized (Figures 3 and 4). Previous studies have produced a systemic *Th* Cre-inducible, DTR-fl/stop/fl DT-inducible ablation, showing that this model’s effects cannot cross the blood-brain barrier and ablating only peripheral nerves (69). Although considered a robust sympathetic marker, TH converts upstream l-tyrosine into l-DOPA. Therefore, a limitation of this method is that it broadly targets all dopaminergic neurons and dopamine-expressing cells, including pancreatic α cells. To address sympathetic specificity, we targeted DBH, which lies downstream of TH and converts dopamine to norepinephrine. To achieve localized effects, we optimized a protocol, ultimately arriving at multiple low-volume intrapancreatic injections. In addition to validating the reduction in sympathetic innervation using 3D clearing and imaging techniques, we confirmed that our ablation model was nontoxic by observing grossly normal behavior and health measures after ablation (Supplemental Figure 3). Despite the overtly efficient and specific ablation of sympathetic nerves throughout the PDAC tumors and tumor-adjacent pancreas, a small portion of TH-staining nerves remained, potentially representing remnants of non-functional neurons. Further functional studies would be required to determine the extent of the activity of the remaining sympathetic signaling. Altogether, this nontoxic model successfully establishes a reliable, sympathetic-specific approach, introducing a novel tool to explore the local impact of sympathetic innervation in PDAC and other cancer models.

To investigate the specific influence of sympathetic nerves on PDAC, we used our sympathectomy method in a slow-growing KPC orthotopic tumor model. In vivo sympathetic nerve studies have primarily been performed using genetically engineered mouse models. We applied our sympathectomy model to orthotopic KPC tumors, characterized by rapid growth, an intact immune system, dense desmoplastic stroma, and limited responsiveness to administered treatments (70). We observed significantly smaller KPC tumor sizes in sympathectomized females, whereas male mice showed no difference (Figure 4). We note that the 6419c5 KPC cells were originally derived from female mice; therefore, there is minimal concern about cross-reactivity in these experiments. We note that autonomic regulation and downstream physiological effects can differ between males and females, which may contribute to the differential tumor susceptibility following sympathetic ablation.

Overall, our in vivo ablation results suggesting intriguing links between biological sex and sympathetic signaling in PDAC raise questions about how sex hormones interact with sympathetic nerves in the disease. Prior studies have shown that males exhibit higher sympathetic activity in certain peripheral tissues, including adipose tissue, influencing metabolic and stress-response pathways (71). Further studies have shown that males tend to have higher sympathetic activity, while females typically have higher parasympathetic activity, and that there are measurable sex differences in peripheral nerve injury responses (72). Sympathetic innervation also shapes immune function in a sex-dependent manner (73), and sex hormones further regulate immune and inflammatory signaling, including pain-related pathways (74). Females typically display a more pronounced inflammatory response, influenced in part by reduced androgen levels (75). Notably, a recent study reported that higher peritumoral β_2_-adrenergic receptor staining was associated with significantly poorer survival, specifically in female PDAC patients, suggesting a sex-specific susceptibility in the context of sympathetic signaling (10). These converging findings underscore the need for future larger-scale studies to rigorously define sex as a biological variable in PDAC-neuron interactions. Leveraging more sensitive tools, such as the chemo-genetic ablation model we generated, could provide critical insights into overall and distinct shifts in cellular composition, signaling pathways, and spatial interactions within the TME.

We demonstrated that CAFs closely surround sympathetic nerves in both murine and human PDAC (Figure 2). To better understand the interactions between sympathetic nerves and CAFs, we used SCG explants to investigate bidirectional paracrine signaling between these cell types. Although SCGs do not directly innervate the pancreas, we selected SCGs for our in vitro modeling studies because they are composed predominantly of postganglionic sympathetic neurons and provide a consistent, well-characterized source of adrenergic input by releasing norepinephrine (76) and some neuropeptide Y (NPY). No cholinergic characteristics were previously observed in these ganglia (77). It is noteworthy that celiac ganglia (CGs), which more directly innervate the pancreas, release a wider range of neurotransmitters, including norepinephrine, NPY, calcitonin gene–related peptide (CGRP), and nitric oxide (78). However, recent evidence suggests that CGs feature both noradrenergic and cholinergic characteristics by expressing somatostatin (SST) and vasoactive intestinal peptide (VIP), as neurons extending from these ganglia are specialized for gut regulation (77).

Coculturing activated pancreas-derived fibroblasts with primary SCG explants revealed changes in the transcriptional programs of the activated PSCs that may directly contribute to tumor progression (Figures 5 and 6). Because CAF subtype specification is shaped by both cell-of-origin and microenvironmental cues, our results suggest that sympathetic nerves serve as niche signals that influence fibroblast activation and fate. PSCs, which already exhibit a partial neuronal-like transcriptional program, may be particularly receptive to sympathetic neural cues, enabling neural signaling to promote iCAF differentiation and ECM remodeling, both key features of tumor progression. We found that sympathetic signaling activates these pancreas-derived CAFs through IL-6/JAK/STAT3, Notch, TGF-β, and TNF-α pathways. We also observed strong enrichment of ECM remodeling programs, a hallmark of CAF-mediated support of PDAC progression (49). Several of the upregulated genes are associated with poor survival of patients with PDAC. Importantly, multiple sympathetic signaling–induced genes encode ligands released into the TME, most notably the axon guidance cue Sema3c, which bind to receptors such as NRP1 and NRP2, which we show are associated with poor PDAC outcomes. SEMA3C upregulation is known to drive EMT in prostate cancer (51), and in PDAC it is instrumental in tumor growth and metastasis, enhancing proliferation and suppressing apoptosis through ERK1/2 activation (79). Together, these findings highlight how sympathetic signaling directly reprograms pancreas-derived CAFs toward tumor-supportive functions, reinforcing a feedforward loop within the TME. Future work could spatially resolve these receptor-ligand interactions between CAFs and sympathetic nerves in vivo and expand understanding of other TME interactions such as immune infiltration.

Our data supported that CAFs can substantially alter SCG transcription and contribute to nerve injury response (Figure 8). Although nerve bodies typically do not divide and thus would not be expected to express cell cycle genes, previous studies suggest that upregulation of cell cycle, immune response, metabolism, and neuron plasticity genes are often linked to peripheral nerve injury (53–56). It is important to note that plated SCG explants are intrinsically in an injury-response state due to their displacement from their native environment (80). However, we ensured even plating in all conditions and reduced stress on neurons by keeping them in their explant form. Our transcriptional observed shift suggests that CAFs may further contribute to neuronal stress and injury responses in PDAC. We also noted that CAFs derived from the pancreas (PSCs) exhibited a more pronounced transcriptional effect than the control fibroblasts, MEFs. Upon closer examination, we also found that the transcriptional influences are markedly different, with only 7.8% overlap between SCG explants cocultured with PSCs versus MEFs. These findings pose interesting questions about whether pancreas-derived CAFs induce a stronger nerve injury response in sympathetic nerves, facilitating their activation and reorganization during PDAC growth. A limitation of our system is its focus on paracrine crosstalk rather than direct cell-to-cell interactions, which may underestimate the extent of bidirectional transcriptional regulation. Single-cell studies could help further distinguish differences in paracrine and physical interactions between SCGs and CAFs, offering deeper insight into their dynamic interplay in PDAC.

In summary, our study highlights neuro-associated survival trends in patients with PDAC and introduces a novel sympathectomy murine model, which could serve as a powerful tool for investigating sympathetic innervation across diverse tumor models. By delineating the intricate interactions between sympathetic nerves and CAFs, we uncovered potential drivers that mediate the bidirectional communication. This work provides a framework for elucidating a deeper understanding of nerve-TME dynamics in PDAC and their contributions to tumor progression, paving the way for more effective, targeted therapeutic strategies.

## Methods

### Sex as a biological variable

Generated survival curves represented both male and female patients. Experiments examined male and female animals, and sex-dimorphic effects are reported and discussed.

### Survival curve generation

We used survival data from 208 deidentified patients diagnosed with PDAC between 2004 and 2020 (39). Kaplan-Meier plots and statistics were derived using R version 3.6.3 and the “survival” package version 3.3.1. A single primary PDAC tumor sample underwent bulk RNA-seq. Tumor high versus low expression of the selected gene was stratified using a threshold that maximized the log-rank *P* value, and the results were reported using the log-rank test. Each stratum contained at least 10 patients, and significance was assessed using a *P* value of less than 0.05. Benjamini-Hochberg FDR correction was performed to calculate adjusted *P* values.

### Murine breeding pairs for SCG dissections

Wild-type C57BL/6J (The Jackson Laboratory, 000664) female mice (aged 2–8 months) underwent 3–5 rounds of breeding to male wild-type mice (aged 2–12 months). Postnatal day 1 to day 4 mice were euthanized in accordance with our IACUC protocol and used for SCG coculture experiments.

### Generation of sympathectomy genetic cross

Homozygous C57BL/6-*Gt(ROSA)26Sor^tm1(HBEGF)Awai^*/J (The Jackson Laboratory, 007900) mice were crossed with heterozygous B6.Cg-*Dbh^tm3.2(cre)Pjen^*/J (The Jackson Laboratory, 033951) mice, generating *Rosa26*-iDTR/*Dbh*-Cre and *Rosa26*-iDTR/*Dbh*-WT offspring. Male and female mice were used, surgeries were conducted between 14 and 16 weeks of age, and tumor growth ranged between 28 and 32 days.

### Cell culture

Murine control mouse embryonic fibroblasts (NIH 3T3, ATCC CRL-1658), immortalized murine PSC cell line (mPSC1; ref. 20), and 6419c5 cells (CVCL_YM21, provided by Ben Z. Stanger, University of Pennsylvania, Philadelphia, PA, USA) were cultured in Dulbecco’s modified Eagle medium (DMEM; Gibco, 11965-092) with 10% fetal bovine serum (FBS; Gibco, Thermo Fisher Scientific Inc.) and 1% penicillin/streptomycin. Cells were maintained at 37°C in a humidified incubator containing 5% CO_2_ and split at 80% confluence for no more than 25–30 passages. Cell lines were routinely tested for mycoplasma.

### Diphtheria toxin–inducible sympathectomy

To validate the sympathectomy using diphtheria toxin (DT; Cayman Chemical, 19657), we initially performed systemic sympathectomies, delivering 500 ng DT via intraperitoneal injection (*n* = 3 *Rosa26-*iDTR/*Dbh*-WT; *n* = 3 *Rosa26*-iDTR/*Dbh*-Cre). Healthy pancreata were collected and fixed 2 weeks later. For validation of pancreas-specific sympathectomy, we injected 20 μL of DT into 4 sites along the pancreas. We tested three DT total concentrations: low, 100 ng (*n* = 1 *Rosa26-*iDTR/*Dbh*-WT; *n* = 1 *Rosa26*-iDTR/*Dbh*-Cre); mid, 250 ng (*n* = 2 iDTR/*Dbh*-WT, Cre^+^; *n* = 2 *Rosa26*-iDTR/*Dbh*-Cre); and high, 500 ng (*n* = 2 *Rosa26-*iDTR/*Dbh*-WT; *n* = 2 *Rosa26*-iDTR/*Dbh*-Cre). Pancreata were collected and fixed 4 weeks after DT injections. Both male and female mice were used for these validations, and ablations were confirmed by 3D immunofluorescent staining.

### Orthotopic PDAC transplant/allograft model

Animals were anesthetized, and 4 intrapancreatic DT injections (4 × 20 μL DT, totaling 500 ng) were delivered. Next, 2.5 × 10^3^ 6419c5 cells in 50% 1× phosphate-buffered saline (PBS) and 50% Matrigel Matrix (Corning, 356231) were injected into the pancreas. Between days 28 and 32 after transplantation, animals were euthanized and perfused with 1× PBS and then with 4% paraformaldehyde (PFA). Tumors, tumor-adjacent pancreas, liver, lungs, spleen, and colon were carefully excised, weighed, and fixed overnight in 4% PFA and transferred to 70% ethanol at 4°C for storage.

### SCG dissections and cocultures

Superior cervical ganglia (SCGs) were extracted as described by Jackson and Tourtellotte (81). Under a dissecting microscope, SCGs were located medial to the bilateral carotid arteries, identifiable by their translucent color and large nerve branch on the superior portion of the ganglia. Using fine forceps, both SCGs were carefully extracted and placed in cold 1× Hanks balanced salt solution (Gibco, 14170-112). Excess tissue was removed from the ganglia. SCGs were dissociated with collagenase type 2 (Worthington Biochemical, LS004174) for 30–35 minutes at 37°C and inverted every 10 minutes, then washed in DMEM (Gibco, 11965-092) containing 10% FBS (Gibco, Thermo Fisher Scientific Inc.), 1% penicillin/streptomycin, and 5 ng nerve growth factor (NGF 2.5S, Gibco, 2402212). Using a fiber-polished glass pipette, SCGs were triturated until the desired explant size was reached. The SCG explants were carefully plated into wells coated overnight at 37°C with a mixture of 100 μg/mL poly-d-lysine hydrobromide (MP Biomedicals, 0210269480) and 10 μg/mL laminin (Thermo Fisher Scientific Inc., 23017015). Thirty minutes before plating of SCG explants, wells were washed once with sterile water and dried at 37°C. SCG explants were either cultured directly together on the same surface or separated by an insert.

### SCG explant cultures for proliferation assays

Between 10 and 20 explants were plated in a coated 24-well plate and allowed to grow for about 70 hours. SCG explant–conditioned medium was transferred to proliferation assays.

### Axon outgrowth coculture

Between 4 and 6 explants were plated per 6-well plate. 10.0 × 10^3^ immortalized cells were added simultaneously to cell culture inserts (Falcon, 353090) and incubated for 70 hours. Phase-contrast imaging captured axon outgrowth.

### RNA-seq coculture

Between 8 and 13 explants were plated in coated 6-well plates and grown with 10.0 × 10^3^ immortalized cells on inserts (Falcon, 353090) for about 70 hours. RNA was isolated using the RNeasy Kit (QIAGEN, 74104).

### In vitro cell staining

Cells were fixed with 4% PFA, washed 3 times with PBS, and blocked in 8% bovine serum albumin (BSA). Cells were incubated overnight with primary antibodies (Supplemental Table 1) in 1% BSA at 4°C, then washed 3 times in 1× PBS for 5 minutes each. Secondary Alexa Fluor–conjugated antibodies in 1% BSA were added in 1% BSA for 2 hours (Supplemental Table 1) at room temperature. Cells were washed 3 times for 5 minutes in 1× PBS and stained with DAPI (Thermo Fisher Scientific Inc., 62248, lot XL3789541) at 1:1,000. Cells were imaged in fresh 1× PBS.

### Immunofluorescent staining of FFPE tissue sections

Sectioned formalin-fixed, paraffin-embedded (FFPE) tissues were deparaffinized and rehydrated through a xylene and ethanol series, finishing with 1× PBS. Upon antigen retrieval with Antigen Unmasking Solution (Vector Laboratories, H-3300) in a high-pressure cooker for 15 minutes, sections were washed and blocked for 30 minutes in 8% BSA at room temperature. Unconjugated primary antibodies (Supplemental Table 1) were applied in 1% BSA and incubated overnight at 4°C. Upon washing 3 times for 5 minutes with 1× PBS, secondary antibodies (Supplemental Table 1) diluted in 1% BSA were added for 2 hours. Sections were washed, quenched using the TrueVIEW reagent (Vector Laboratories, NC202386), and stained with DAPI (Thermo Fisher Scientific Inc., 62248, lot XL3789541) 1:1,000. Sections were washed and then mounted using ProLong Gold antifade reagent (Life Technologies Corp., P36930).

### iDISCO

iDISCO was performed on sections of KPC orthotopic tumors, as described by Renier et al. (82) and the associated protocol (https://idisco.info/). Fixed tumors were sectioned into 2- to 3-mm slices. Samples were first pretreated with methanol, delipidated in dichloromethane (Sigma-Aldrich, 270997-12X100ML), bleached in 5% H_2_O_2_ in methanol, rehydrated in a decreasing-concentration methanol series, and then washed with the PTx.2 mix (10× PBS and Triton X-100 [Sigma-Aldrich, X100-500ML]). Next, samples were permeabilized for 2 days (PTx.2, glycine [Sigma-Aldrich, G7126-500G], and dimethyl sulfoxide) and blocked (PTx.2, donkey serum [Jackson ImmunoResearch Labs, 017-000-12], and dimethyl sulfoxide). Samples were washed with PTwH solution (10× PBS, Tween 20 [Sigma-Aldrich, P9416-100ML], and heparin [Sigma-Aldrich, H3393-50KU]) and incubated with the primary antibody (Supplemental Table 1) for 4 days. After additional washes in PTwH solution, tissues were incubated for 4 days with secondary antibodies (Supplemental Table 1). Upon PTwH washes, samples underwent a methanol dehydration series. Lastly, samples were transferred to dibenzyl ether (DBE) (Sigma-Aldrich, 108014-1KG) and mounted in DBE on 2-sided 1.5 μm coverslip chambers constructed from a thin silicone sheet with a round cutout, secured using silicone glue.

### EZ Clear

The EZ Clear protocol was performed on tumor-adjacent pancreata and 2- to 3-mm tumor sections, as described by Hsu et al. (83). Samples were rehydrated in 1× PBS and delipidated in a 50% tetrahydrofuran (MilliporeSigma, 186562) and 50% water mixture for 2 days, then washed with 1× PBS and blocked (1× PBS, Triton X-100, donkey serum, and sodium azide [MilliporeSigma, S2002]) for 2 more days. Primary antibodies (Supplemental Table 1) were incubated in blocking solution for 3 days. Samples were washed with 1× PBS, and the secondary antibody (Supplemental Table 1) was added and incubated for 3 days. Tissues were then washed with 1× PBS, and EZ Clear solution was added (sodium phosphate, urea [MilliporeSigma, U5378], sodium azide [MilliporeSigma, S2002], and Nycodenz [Accurate Chemical & Scientific, 100334-594]) overnight. Fresh EZ Clear solution was used to mount tissues between 2-sided 1.5 μm coverslip chambers constructed from a thin silicone sheet with a round cutout, secured with silicone glue.

### Picro Sirius Red staining

Collagen fibers were visualized using the Picro Sirius Red Stain Kit (Abcam, ab150681). FFPE sections were deparaffinized and rehydrated through the following graded series: 3 × 5 minutes in 100% xylene, 2 × 2 minutes in 100%, 2 × 2 minutes in 95%, 1 × 2 minutes in 70% ethanol; and rinsed in distilled water. Sections were incubated for 2 hours with 150 μL of Picro Sirius Red solution at room temperature on a rocker, and then 200 μL of the acetic acid solution was applied twice in 4-minute incubations. Sections were then dehydrated in 95%, then 100% ethanol, and cleared in 100% xylene (4 minutes each). Coverslips were mounted using resin-based mounting medium (Permount, Electron Microscopy Sciences, 17986-01).

### Confocal microscope

Cleared tissues were imaged using an inverted laser-scanning confocal microscope with Airyscan.2 (Zeiss, LSM 980) and a ×10 air objective. Tiles were stitched and quantification was completed using Zen microscopy software (Zeiss).

### ApoTome3

Stained FFPE tissues and cells were imaged using the ApoTome3 grid-based optical sectioning microscope (Carl Zeiss). ×10 and ×20 air objectives were used. Zen microscopy software automated tile fusion and quantification.

### AxioScan slide scanner

Whole-slide scanning was performed using the Zeiss AxioScan 7 Slide scanner. The automatic scanner acquired ×20 tiled images of tissue sections. Automated tile processing fused images.

### Cytation 5

Explant axon outgrowth was imaged using digital phase contrast and was acquired using the BioTek Cytation 5. The microscope incubator was set at 37°C, and automated exposure and focusing were used. Outgrowth was quantified using ImageJ (NIH) tracing and quantification (8+ random, equally distributed locations).

### IncuCyte live-cell analysis systems

For wound healing assays, we used the IncuCyte system from Sartorius wound-healing program (×10, phase contrast).

### Proliferation assay

The CellTiter 96 AQueous One Solution Cell proliferation Assay (MTS) (Promega, G3580) was used to assess cell proliferation. 2 × 10^3^ mPSC1 or 6419c5 cells per well were plated in a 96-well plate approximately 20 hours before conditioned medium was added. The growth media were replaced with conditioned and control media, and the cells were allowed to grow for approximately 30 hours. As directed by the manufacturer’s protocol, CellTiter 96 AQueous One Solution was added and incubated at 37°C for 4 hours. Absorbance was recorded at 490 nm with a plate reader. Blank-well absorbance was subtracted from the experimental absorbance results.

### Wound healing assay

2.5 × 10^3^ mPSC1 or 7 × 10^3^ 6419c5 cells were plated in a 96-well Incucyte Imagelock plate (Sartorius). At 90%–100% confluence, mitomycin C (10 ng/mL) was added and incubated at 37°C for 2 hours. Cells were then carefully washed with 1× PBS (with Ca^2+^ and Mg^2+^). Wells were scratched with a uniform cell scratcher, treatments were applied, and cells were placed in the IncuCyte system for scanning every 3 hours. Quantification was performed using ImageJ. Recombinant Sema3c (murine, R&D Systems, 1728-S3; and human, R&D Systems, 5570-S3) was administered at 0.1, 0.5, 1, and 2 μg/mL.

### Cell adhesion assay

The CytoSelect 48-well Cell Adhesion Assay (ECM Array, Colorimetric, Cell Biolabs, CBA-070) was used to quantify cell adhesion. The manufacturer’s protocol was followed. 0.357 × 10^5^ mPSC1 cells (control and SCG medium–treated) were added and incubated for 90 minutes at 37°C. Cells were carefully washed, treated with the Cell Stain Solution, washed again, and then treated with the Extraction Solution. Absorbance was recorded at 560 nm with a plate reader.

### Cytokine array

The Proteome Profiler Mouse XL Cytokine Array (R&D Systems, ARY028) protocol was followed as described by the manufacturer. Membranes were prepared and each treated with conditioned medium filtered with Amicon Centrifugal Filter Units, 50 kDa. Blots were imaged using a chemiluminescence imager, quantified with ImageJ, and plotted using Morpheus (https://software.broadinstitute.org/morpheus/).

### Bulk RNA-seq: RNA preparation, library preparation, and sequencing

Total RNA was purified using the RNeasy Kit (QIAGEN, 74004). β-Mercaptoethanol was used as the reducing agent, and RNA extraction was performed according to the QIAGEN protocol. RNA concentration and integrity were verified using a bioanalyzer, and 50 million read pairs per library were prepared and sequenced by the Massively Parallel Sequencing Shared Resource at Oregon Health & Science University (OHSU).

### Data alignment and preprocessing

Sequencing data alignment and quantification were performed with STAR (v2.7.10b) (84). FASTQ files containing sequencing reads for each sample were aligned to the mouse genome assembly GRCm39. STAR alignment options were set to standard ENCODE parameters as detailed in the STAR manual. Gene-level quantification was performed by setting STAR quantification mode to “GeneCounts” and used the GENCODE primary assembly basic genome annotation (M33) (85). Stranded reads for each gene were collected into separate counts matrices representing PSC and nerve samples.

### RNA-seq analysis

All post-alignment RNA-seq data analysis and visualization were performed in R (v4.2.2) (86). For differential expression analysis, the standard DESeq2 (v1.38.3) (87) workflow was applied to PSC samples and nerve samples separately. DESeq2 two-factor modeling was used to compare experimental groups while accounting for the experimental batch as a covariate (6/5/2023, 7/5/2023, 8/11/2023, and 9/5/2023). Genes whose read count was cumulatively less than 10 across samples were excluded from differential expression testing in addition to the default DESeq2 low read count gene filter. PCA used read counts that were modified by DESeq2 variance-stabilizing transformation (VST) and subsequently batch-corrected with limma (v3.54.2) (88). Heatmap visualizations of DESeq2 results were generated with pheatmap (v1.0.12) using VST batch-corrected counts and set to scale data by row.

### Gene set enrichment analysis

Differentially expressed genes from the DESeq2 analysis were input into the GSEA Molecular Signatures Database (MSigDB) gene set enrichment analysis (https://www.gsea-msigdb.org/gsea/msigdb). Mouse molecular signatures collections, including hallmark gene sets (MH) and GO biological processes (M5, BP), were used to evaluate categorization.

### Statistics

Sample sizes and replicates are reported. Statistical analyses were primarily performed using GraphPad Prism software [version 10.4.0 (527)]. The unpaired 2-tailed *t* test was used to compare parametric data between 2 groups. ANOVA was performed for multiple conditions. *P* < 0.05 was considered statistically significant.

### Study approval

#### Human tissue samples.

Human PDAC patient tissue samples and informed consent were obtained from the OPTR (Institutional Review Board approved, STUDY00003609). All methods were carried out in strict compliance with the ethical regulations of the Institutional Review Board and the institution. Samples were derived from FFPE primary tumors that underwent pathological review. Tumor-rich areas were macrodissected for nucleic acid extraction and RNA-seq. Pathology review was performed at OHSU, and RNA extraction and sequencing were conducted by Tempus (GEO accession GSE205154).

#### Animal studies.

Studies were approved by the Institutional Animal Care and Use Committee (IACUC) at Oregon Health & Science University (OHSU), which reviewed and oversaw each animal model experiment in accordance with NIH guidelines for the humane treatment of animals.

### Data availability

All sequencing data from this study were deposited in the publicly available Gene Expression Omnibus database (GEO GSE318481; https://www.ncbi.nlm.nih.gov/geo/query/acc.cgi?acc=GSE318481).

## Author contributions

ALS conducted all experiments described in this article with support from PD, JG, TK, BC, and MJK. KH analyzed the bulk RNA-seq data. CP analyzed survival data from the OPTR with support from RCS. ALS, MHS, DLM, TAZ, and SEE designed the experiments and drew conclusions from the data. SEE supervised all experiments and analyses. ALS and SEE wrote the manuscript with input from all authors.

## Funding support

This work is the result of NIH funding, in whole or in part, and is subject to the NIH Public Access Policy. Through acceptance of this federal funding, the NIH has been given a right to make the work publicly available in PubMed Central.

- NIH grants R01CA257452 (to DLM and TAZ), R01CA250917, and Memorial Sloan Kettering Cancer Center P30CA008748 (to MHS).
- Cancer Early Detection Advanced Research Center (CEDAR), Project ID# Faculty Startup 2023-1768, Project ID# Exploratory Grants 2025-2044 and 2023-1840 (to SEE).
- Oregon Health & Science University, Knight Cancer Institute’s Scientific Operations Collaborative Grant (to ALS).

## Supplementary Material

Supplemental data

Unedited blot and gel images

Supporting data values

## Version 1 02/19/2026

In-Press Preview

## Version 2 04/08/2026

Electronic publication
