# Supplementary material for: Sympathetic nerve–fibroblast crosstalk drives nerve injury, fibroblast activation, and matrix remodeling in pancreatic cancer
Source: JCI Insight. 2026 Feb 19;11(7):e192814. doi: 10.1172/jci.insight.192814 (PMC13134732; doi:10.1172/jci.insight.192814)
Supplement: Supplemental data [file jciinsight-11-192814-s050.pdf]

Supplemental Material

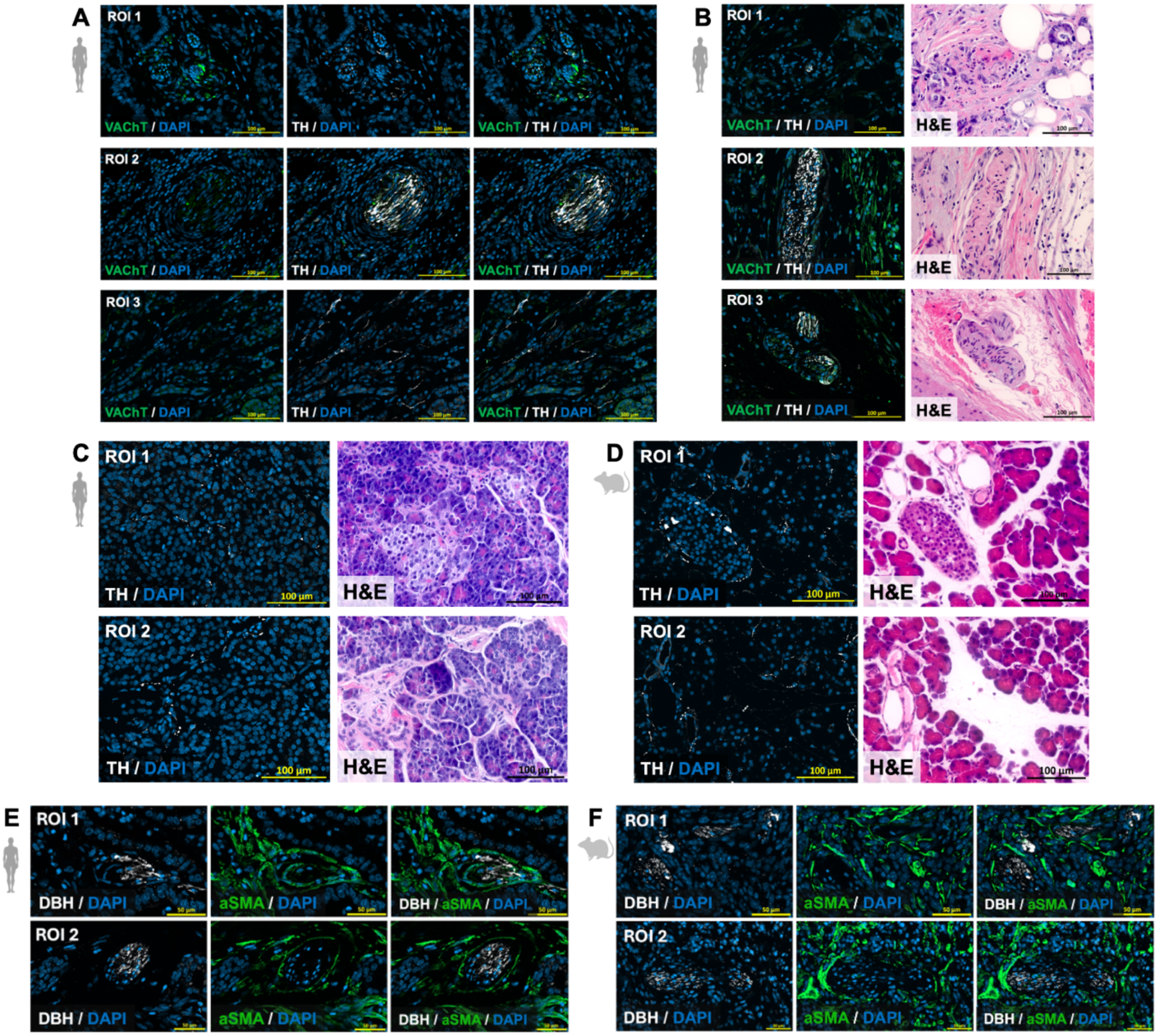

Supplemental Figure 1. Immunofluorescent staining of variable sympathetic innervation in human and murine PDAC tumors. (**A**) Co-immunofluorescent images illustrating diverse patterns of intratumor innervation in human PDAC, including nerve bundles with varying sympathetic and parasympathetic compositions, orientation and quantity. Samples were stained with VAChT (green), TH (white), and DAPI (cyan). Scale bar, 100 μm. (**B**) Co-immunofluorescent images showing intratumor nerve bundles of different sizes in human PDAC. ROI1 depicts a small bundle, ROI2 a large bundle, and ROI3 multiple medium-sized bundles in proximity. Staining includes VAChT (green), TH (white), and DAPI (cyan). Scale bar, 100 μm. Images are shown with matched H&E. Immunofluorescent images of sympathetic innervation in (**C**) human and (**D**) murine tumor-adjacent pancreas. ROI1s highlight innervation of acini and islets of Langerhans,

and ROI2s show innervation of ducts and interlobular connective tissue. Scale bars, 100 µm. Co-immunofluorescent images of intratumor sympathetic nerves in (**E**) human PDAC and (**F**) murine KPC tissues. Two regions of interest (ROIs) show intratumor nerve bundles stained with the sympathetic nerve marker, DBH (white), α-SMA (green), and DAPI (cyan). Scale bars, 50 µm.

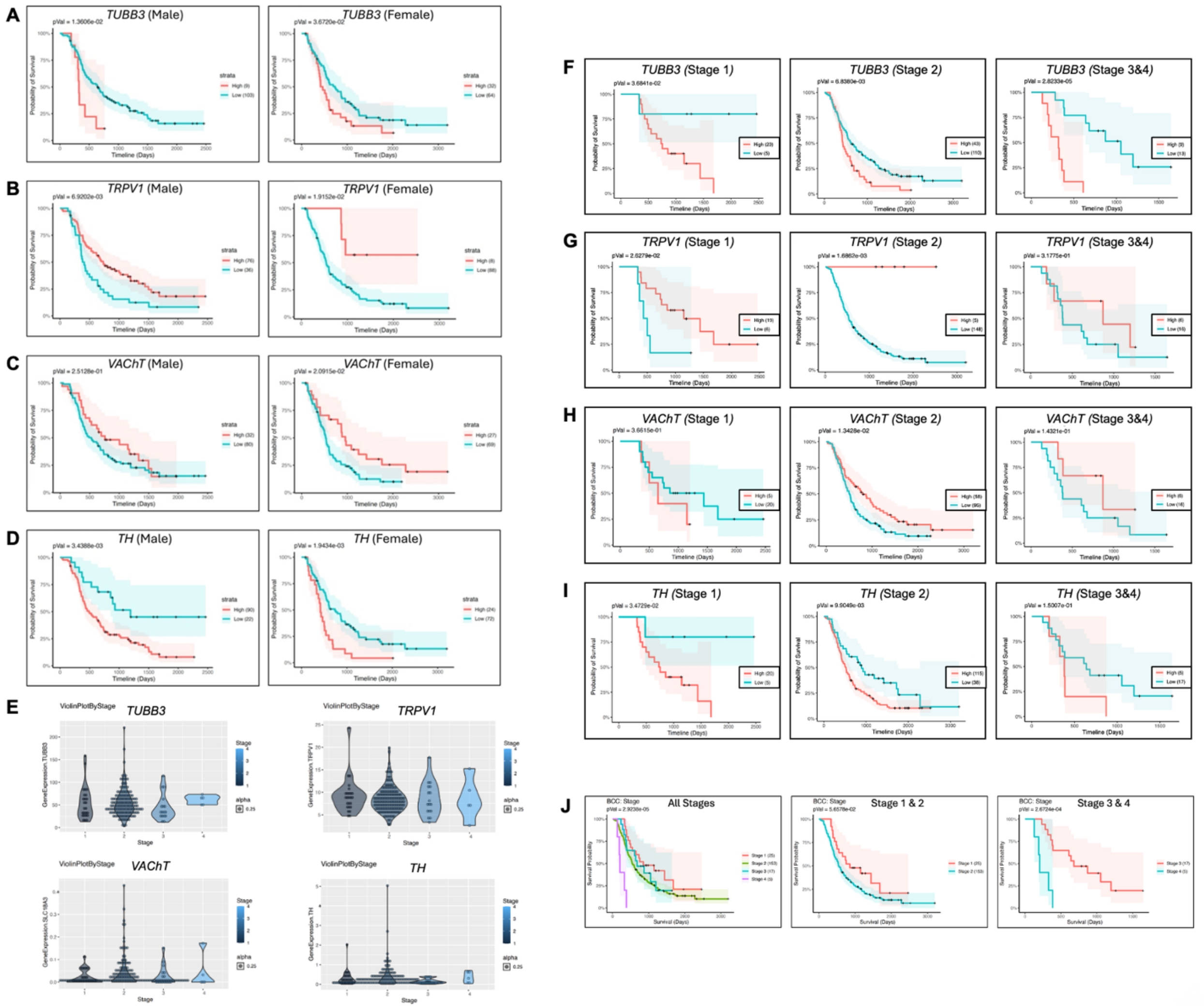

Supplemental Figure 2: Kaplan-Meier estimates of PDAC survival stratified by sex and stage. Kaplan-Meier estimates of PDAC patient survival associated with primary tumor high or low gene expression and stratified by sex of: (**A**) *TUBB3*, (**B**) *TRPV1*, (**C**) *VAChT* (*SLC18A3*), and (**D**) *TH*. The red line represents PDAC patients with expression, and the blue line represents patients with low expression (n = 208 total PDAC patients). (**E**) Violin plots of RNA expression by stage of *TUBB3*, *TRPV1*, *VAChT* (*SLC18A3*), and *TH*. Kaplan-Meier estimates of PDAC patient survival associated with primary tumor high or low gene expression and stratified by stage 1, 2,

and 3 & 4 combined of (**F**) TUBB3, (**G**) *TRPV1*, (**H**) *VAChT* (*SLC18A3*), and (**I**) TH. The red line represents PDAC patients with high expression, and the blue line represents patients with low expression (stage 1, n = 25; stage 2, n = 153; stages 3 and 4, n = 22). Kaplan-Meier estimates of PDAC patient survival associated with (**J**) all stages (stage 1 (red line), stage 2 (green line), stage 3 (blue line), stage 4 (violet line), followed by plots comparing stage 1 (red line) and 2 (blue line), and stage 3 (red line) and 4 (blue line).

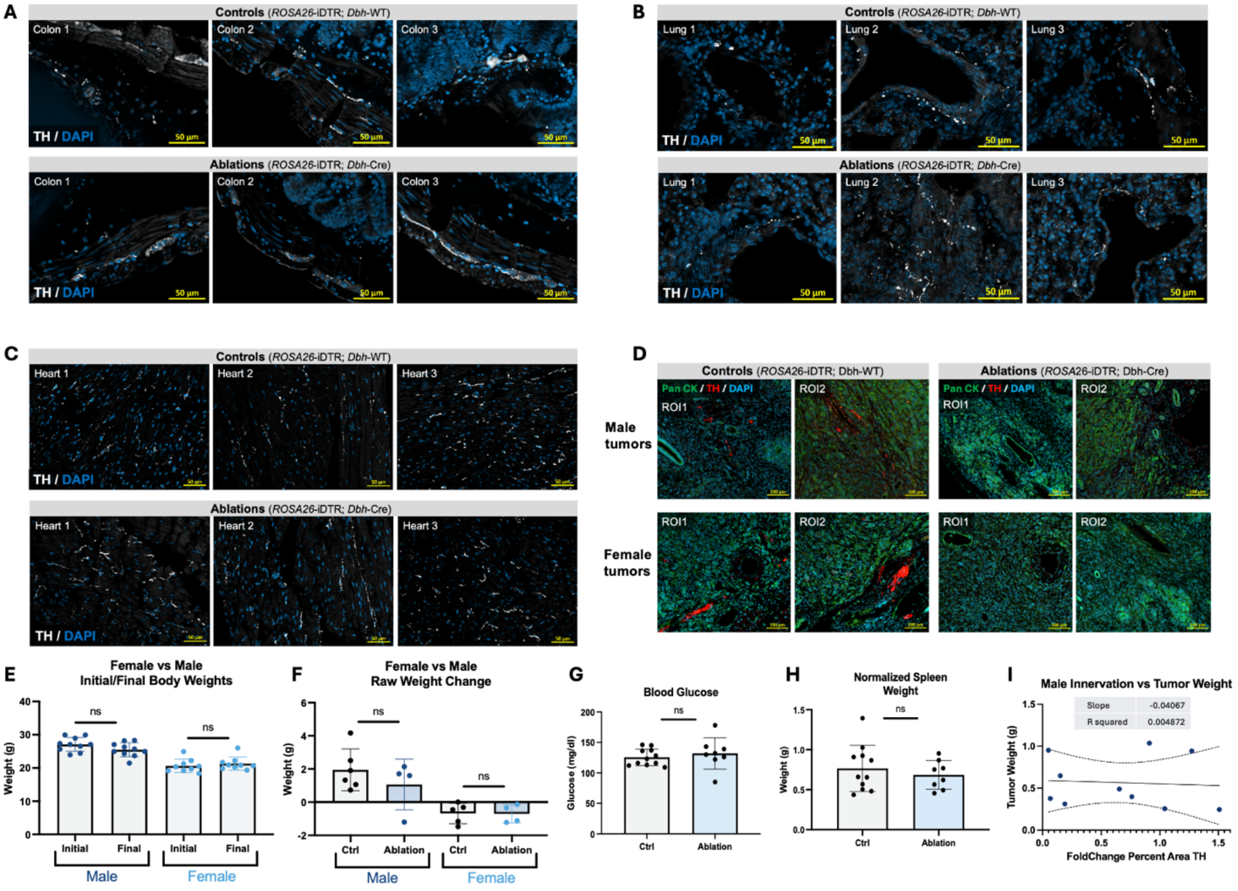

Supplemental Figure 3: Validation of pancreas-specific sympathectomy and systemic physiological assessments. Representative 15 µm maximum-intensity projections of (**A**) colon, (**B**) lung, and (**C**) heart sections stained for TH (white) and DAPI (cyan), demonstrating preserved sympathetic innervation in control and ablated murine off target organs. Scale bars, 50 µm. (**D**) Representative immunohistochemistry staining of intratumor innervation in control and ablated KPC tumors in male and female mice. Samples were stained with pan-CK (green), TH (red), and DAPI (cyan). Scale bars, 100 µm. (**E**) Initial (tumor-free) and final (experimental endpoint) body

weights (g) of female (n = 9) and male (n = 10) mice. (**F**) Raw weight change (g) of control and sympathetic ablated mice (female n = 9, male n = 10). (**G**) Blood glucose levels (mg/dL) of control (n = 11) and sympathetic ablated (n = 9) mice at endpoint. (**H**) Normalized spleen weights (spleen weight / body weight) of control and ablated mice (female n = 9, male n = 9). All data are shown as individual points with mean ± SD; ns indicates $P > 0.05$; unpaired 2-tailed Student's t-test. (**I**) Correlation between male normalized TH percent area compared to KPC orthotopic tumor weights (g).

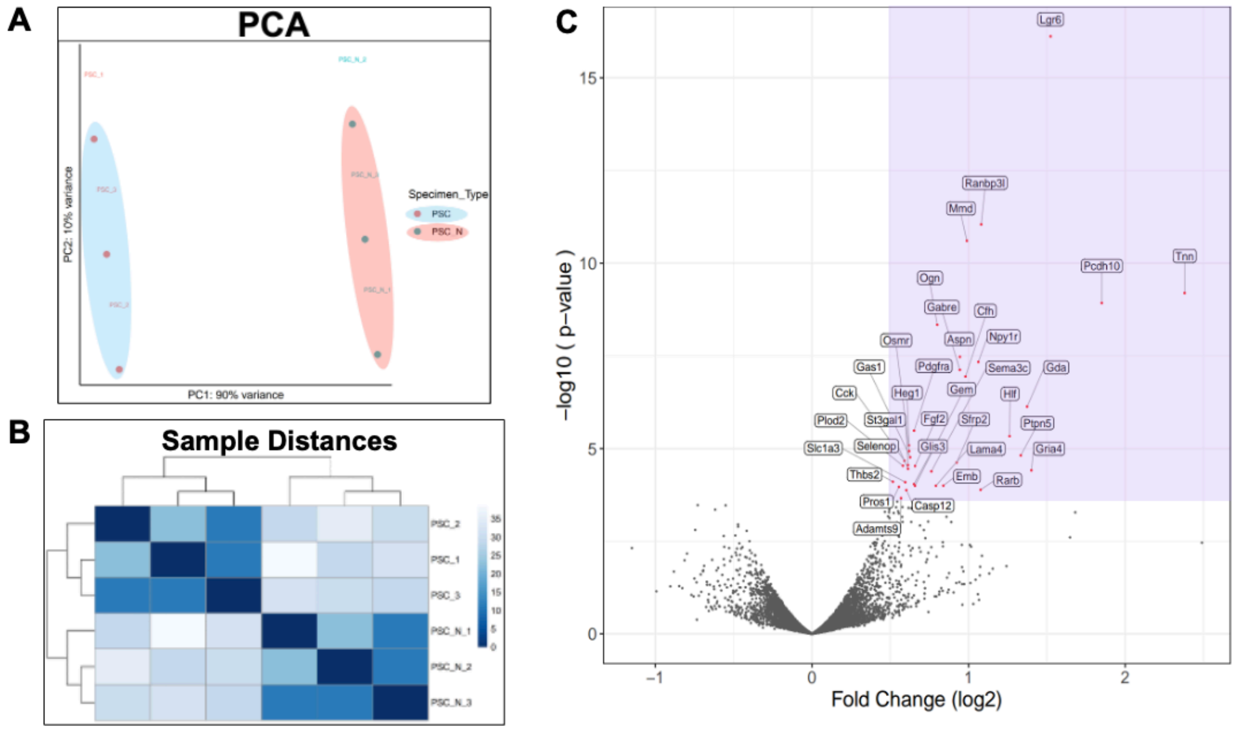

D

Gene	adjusted_p_ values_bh	Survival
ADAMTS9	0.25	NA
ASPN	0.03	Positive
CASP12	0.09	NA
CCK	0.26	NA
CFH	0.06	NA
EMB	0.09	NA
FGF2	0.25	NA
GABRE	0.02	Negative
GAS1	0.05	Negative
GDA	0.11	NA
GEM	0.08	NA
GLIS3	0.18	NA
GRIA4	0.17	NA
HEG1	0.06	NA
HLF	0.00	Positive
LAMA4	0.03	Negative
LGR6	0.14	NA
MMD	0.01	Negative
NPY1R	0.01	Positive
OGN	0.01	Positive
OSMR	0.01	Negative
PCDH10	0.05	Positive
PDGFRA	0.09	NA
PLOD2	0.00	Negative
PROS1	0.03	Positive
PTPN5	0.01	Positive
RANBP31	0.02	Positive
RARB	0.08	NA
SELENOP	0.00	Positive
SEMA3C	0.11	NA
SFRP2	0.14	NA
SLC1A3	0.28	NA
ST3GAL1	0.05	Negative
THBS2	0.09	NA
TNN	0.05	Positive

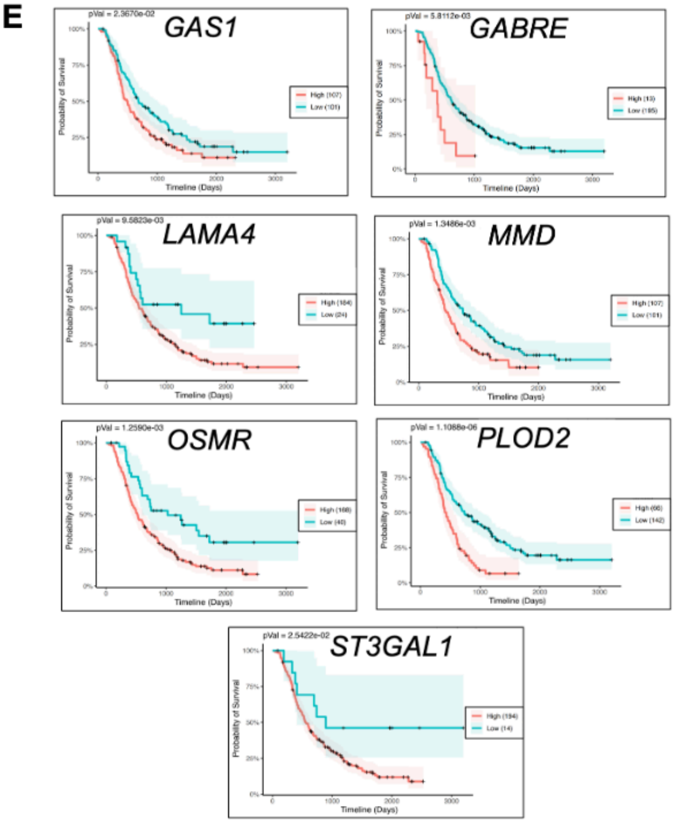

Supplemental Figure 4: PSC bulk RNA-seq. (**A**) Principal component analysis (PCA) showing samples in the PSC condition compared to PSC+SCG (PSC_N) samples. PCA was performed

on the top 500 most variable genes (batch-adjusted VST counts). (**B**) Batch-adjusted sample distances for co-cultured PSC. (**C**) Volcano plot of bulk RNA-seq results showing genes increased in PSC samples (negative log2 fold change, left) and PSC+SCG samples (positive log2 fold change, right) conditions. Purple shades the upregulated protein-coding genes passing the 0.05 adjusted p-value and 0.5 log2 fold change cutoff. (**D**) List of 35 significantly upregulated protein-coding genes in PSCs when co-cultured with SCGs and their associated human PDAC survival. Adjusted *P* values were generated with a Benjamini-Hochberg (BH) FDR correction (n = 208 patients). (**E**) Kaplan-Meier estimates of genes associated with overall poor PDAC patient survival: *GAS1*, *GABRE*, *LAMA4*, *MMD*, *OSMR*, *PLOD2*, and *ST3GAL1*. The red line represents PDAC patients with high gene expression, and the blue line represents patients with low gene expression (n = 208 total).

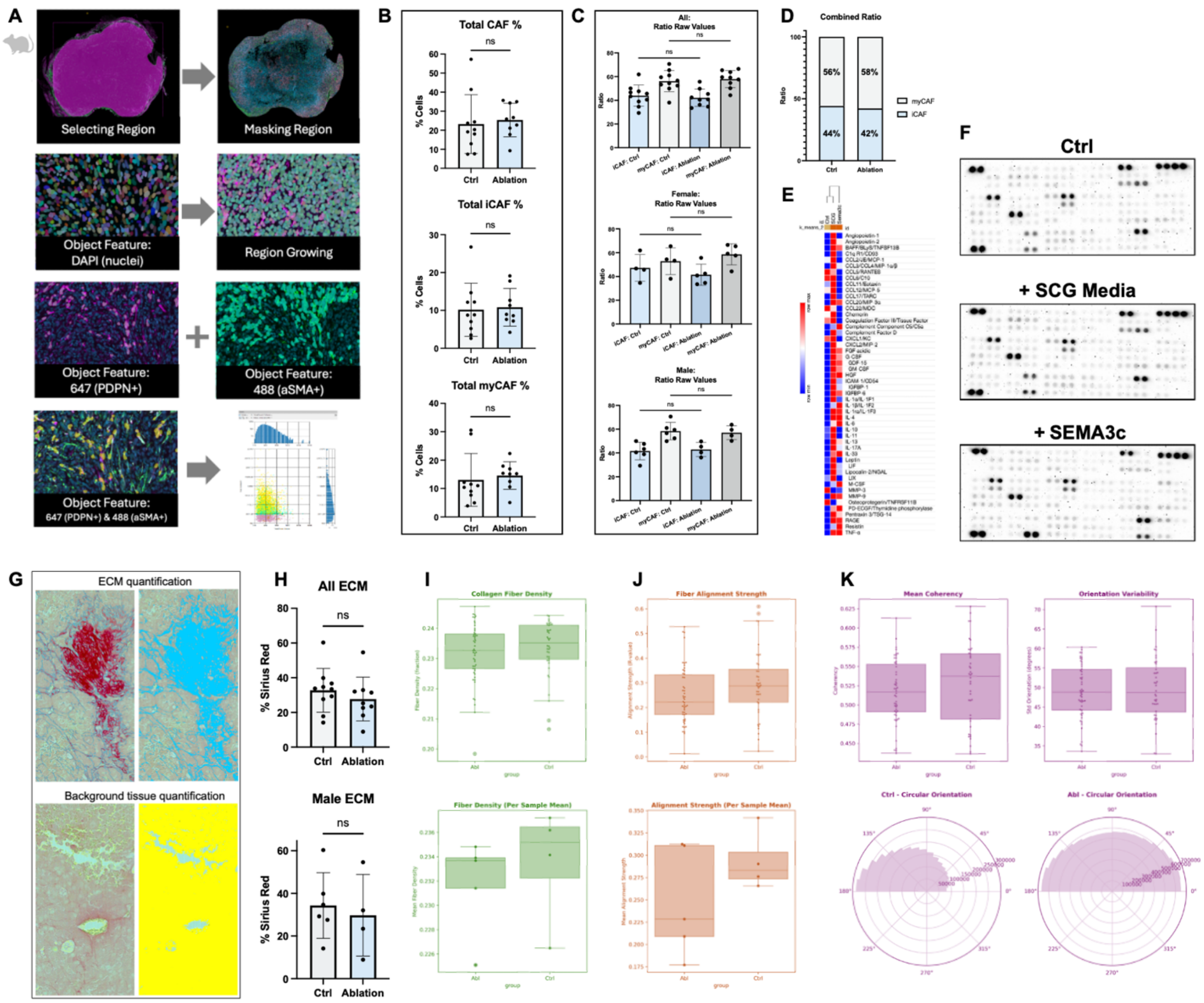

Supplemental Figure 5: CAF and ECM phenotype quantifications. (**A**) Schematic illustrating the CAF phenotype quantification pipeline performed using Zeiss Zen analysis software with the Cellpose plugin. DAPI (blue) outlined object features and CAFs were co-stained with PDPN (magenta) and aSMA (green). myCAFs were quantified as aSMA+/PDPN+ cells, while iCAFs were quantified aSMA-/PDPN+ cells. (**B**) Quantification of CAF populations in control and sympathectomized KPC tumors: total CAF percentage, total iCAF percentage, and total myCAF percentage. (**C**) Ratios of iCAF-to-myCAF in all tumors combined, female mice only, and male mice only. (**D**) Summary of the iCAF-to-myCAF ratio % summary of all tumors combined. (**E**) Heat map of 50 iCAF-associated cytokines measured with the cytokine array. (**F**) Visualization of cytokine array membranes treated with PSC media of PSCs that were previously cultured in control media, + SCG media, and media containing recombinant murine SEMA3C. (**G**) Representative images of ECM and tissue background, with quantification performed using Zen Zeiss analysis. (**H**) All and male tumor ECM quantification. Each dot represents one tumor; data are presented as mean ± SD; ns, $P > 0.05$; unpaired, 2-tailed Student's t-test. (**I**) Biomni quantification of collagen fiber density. (**K**) Biomni quantification of mean coherency, orientation variability and circular variability.

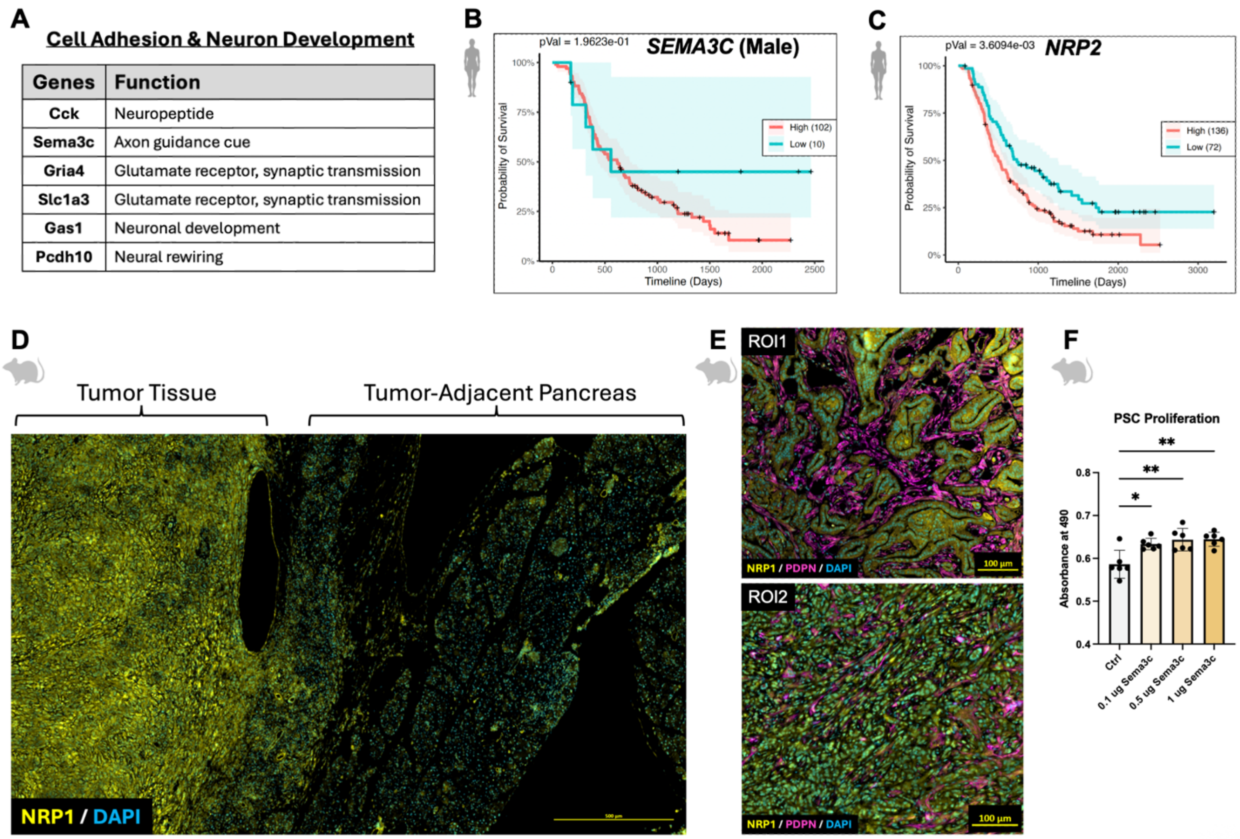

Supplemental Figure 6: SEMA3C and NRP1 additional evaluations. (**A**) List of genes associated with cell adhesion and neuron development. Kaplan-Meier estimates of PDAC patient survival associated with primary tumor high or low gene expression of (**B**) *SEMA3C* (male patients n = 112) and (**C**) *NRP2* (all patients combined n = 208). The red line represents PDAC patients with high expression, and the blue line represents patients with low expression. (**D**) Representative immunofluorescent image of NRP1 (yellow) in murine KPC tumor and tumor-adjacent pancreas. Scale bar, 500 µm. (**E**) Co-immunofluorescent images of NRP1 (yellow) and PDPN (magenta) in a well differentiated (ROI1) and poorly differentiated (ROI2) KPC section. Scale bars, 100 µm. (**F**) Proliferation of PSC cells treated with a gradient of recombinant murine SEMA3C. n = 6 technical replicates, bar plots mean ± SD; *$P < 0.05$, **$P < 0.01$; 2-way ANOVA.

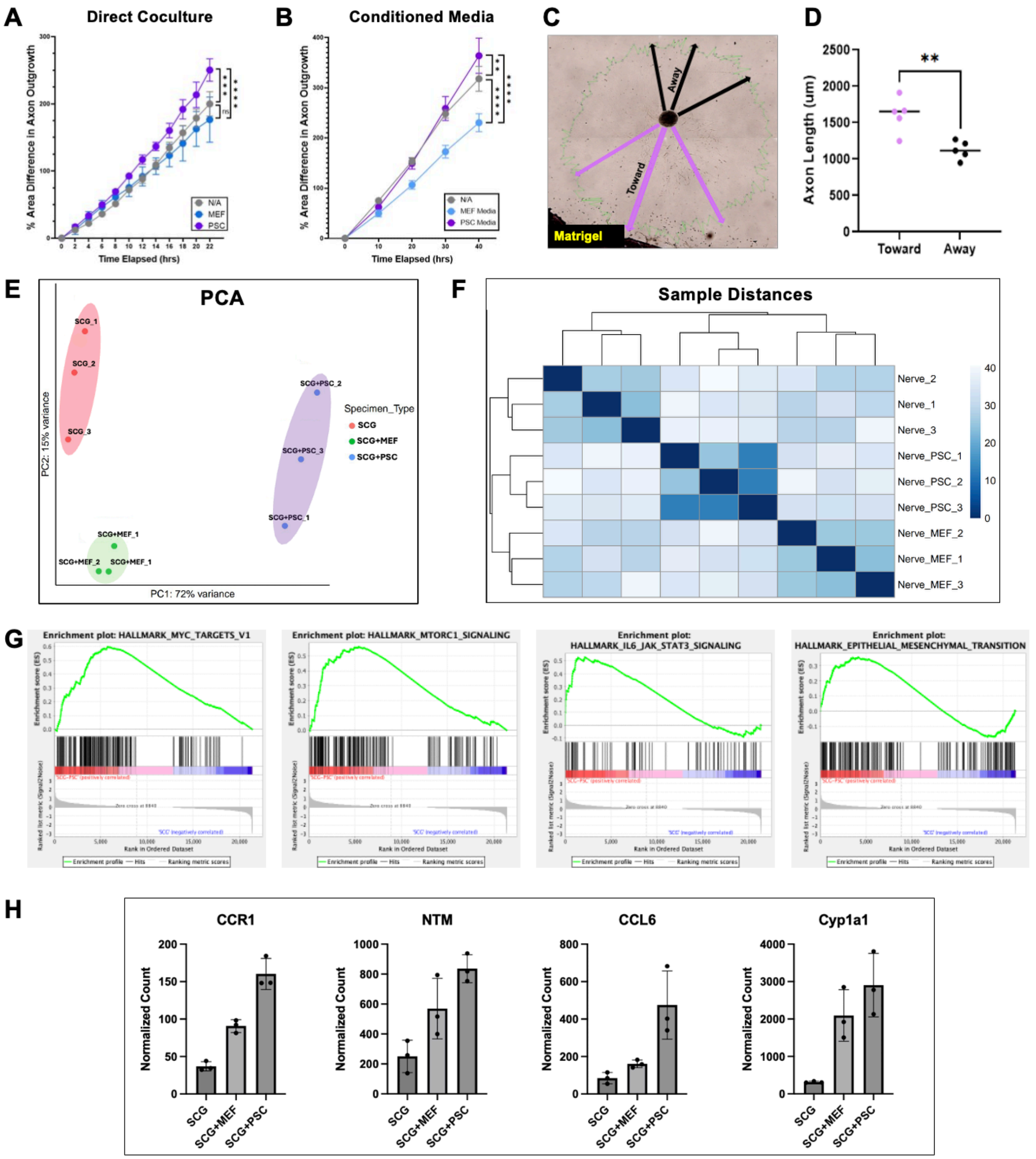

Supplemental Figure 7: SCG radial outgrowth and bulk RNA-seq with GSEA plots. (**A**) Radial outgrowth rate of SCG explants (n = 3 per group) when directly co-cultured with no cells, MEFs, and PSCs. (**B**) Radial axon outgrowth rate of SCG explants (n = 3 per group) when indirectly co-cultured with no cells, MEFs, and PSCs. (**C**) Representative image of directional co-culture. 15 radial measurements, originating at the explant cell body edge, were collected in the quadrant toward and away from the Matrigel dome containing PSCs. (**D**) Averaged directional

outgrowth of explants located within 3 mm of the Matrigel dome containing PSCs. Each point represents one explant (n = 5). Mean ± SEM; ns $P > 0.05$, **$P < 0.01$, and ***$P < 0.001$, ****$P < 0.0001$; unpaired, 2-tailed Student's t test. (**E**) PCA plot representing samples of the SCG, SCG+MEF, and SCG+PSC conditions. PCA was performed on batch-adjusted VST counts for the top 500 most variable genes. (**F**) Batch-adjusted sample distances for SCG co-cultured samples. (**G**) GSEA plots of Hallmark gene sets upregulated in SCG+PSC compared to SCG: MYC Signaling (NES = 2.04, FDR q-value = 0.0), MTORC1 (NES = 1.92, FDR q-value = 0.0), IL6/JAK/STAT3 signaling (NES = 1.64, FDR q-value = 0.003), and EMT (NES = 1.24, FDR q-value = 0.192). (**H**) Normalized counts comparing SCG, SCG+MEF and SCG+PSC transcription of: *Ccr1*, *Ntm*, *Ccl6*, and *Cyp1a1*.

REAGENT	SOURCE	IDENTIFIER	LOT	CONCENTRATION
**Primary Antibodies for Immunostaining**				
VAChT Monoclonal Antibody (N6/38)	Invitrogen	MA5-27662	ZK4530702	1:200
Anti-Tyrosine Hydroxylase	EMD Millipore	AB152	3870479	1:200
Alpha-Smooth Muscle Actin Monoclonal Antibody (1A4 (asm-1))	Invitrogen	MA5-11547	ZA4182968	1:200
Anti-Mo Podoplanin	Invitrogen	14-5381-82	2358694	1:100
Dopamine-beta hydroxylase polyclonal antibody	Thermo Fisher Scientific	PA5-34664	ZG4406531A	1:100
NRP1	Abcam	ab81321	GR3370248-2	1:200
Cytokeratin Pan Type I/II Antibody Cocktail [AE1/AE3]	Invitrogen	MA5-13156	XK3722334	1:200
**Secondary Antibodies for Immunostaining**				
Alexa Flour 555 donkey anti-rabbit IgG (H+L)	Invitrogen	A3172	2482963	1:400
Alexa Flour 488 donkey anti-mouse IgG (H+L)	Invitrogen	A21202	2428531	1:400
Alexa Fluor 647 Goat Anti-Syrian Hamster IgG H&L	Abcam	ab180117	GR3404612-2	1:200

Supplemental Table 1: Antibodies used for immunostaining. The primary and secondary antibodies used for immunostaining experiments, including reagent name, commercial source, catalog number, lot number and working dilution.
