# Supplementary material for: Sympathetic nerve–fibroblast crosstalk drives nerve injury, fibroblast activation, and matrix remodeling in pancreatic cancer
Source: JCI Insight. 2026 Feb 19;11(7):e192814. doi: 10.1172/jci.insight.192814 (PMC13134732; doi:10.1172/jci.insight.192814)

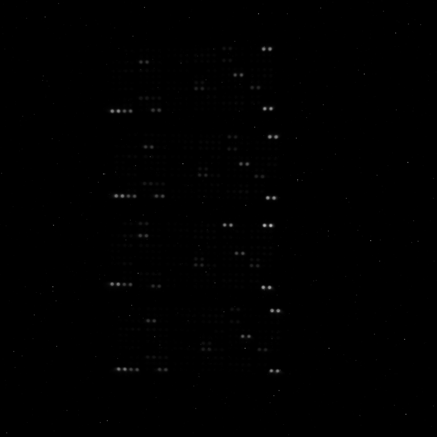

Supplement: Unedited blot and gel images [file jciinsight-11-192814-s051.pdf]
